# Sex differences in child and adolescent physical morbidity: cohort study

**DOI:** 10.1136/bmjpo-2017-000191

**Published:** 2017-12-29

**Authors:** Helen Sweeting, Elise Whitley, Alison Teyhan, Kate Hunt

**Affiliations:** 1 MRC/CSO Social and Public Health Sciences Unit, University of Glasgow, Glasgow, UK; 2 Population Health Sciences, Bristol Medical School, University of Bristol, Bristol, UK

**Keywords:** adolescent health, epidemiology, general paediatrics

## Abstract

**Background:**

Evidence on sex differences in physical morbidity in childhood and adolescence is based largely on studies employing single/few physical morbidity measures and different informants. We describe sex differences in a wide range of parent/carer-reported physical morbidity measures between ages 4 and 13 years to determine evidence for a generalised pattern of an emerging/increasing female ‘excess’.

**Methods:**

Parents/carers (approximately 90% mothers) of the population-based UK ALSPAC cohort provided data on general health, physical conditions/symptoms and infections in their child approximately annually between ages 4 and 13. Logistic regression analyses determined the odds of each morbidity measure being reported in respect of females (vs males) at each age and the sex-by-age interaction, to investigate any changing sex difference with age.

**Results:**

Six measures (general health past year/month, high temperature, rash, eye and ear infections) demonstrated an *emerging female ‘excess’*, and six (earache, stomach-ache, headache, lice/scabies, cold sores, urinary infections) an *increasing female ‘excess’*; one (breathlessness) showed a *disappearing male ‘excess’*. Just two showed either an *emerging* or *increasing male* ‘excess’. Most changes were evident during childhood (prepuberty). Six measures showed *consistent female ‘excesses’* and four *consistent male* ‘*excesses’*. Few measures showed no sex differences throughout this period of childhood/early adolescence.

**Conclusion:**

Sex differences are evident for a wide range of parent-reported physical morbidity measures in childhood and early adolescence. Far more measures showed an emerging/increasing female ‘excess’ than an emerging/increasing male ‘excess’. Further studies are required to examine whether patterns differ across sociodemographic/cultural groups, and to explain this generalised pattern.

**What is already known on this topic?**

- Evidence of an emerging/increasing female ‘excess’ in physical morbidity over the transition to adolescence is less well established than that in respect of psychological morbidity.
- Most studies of sex differences in child/adolescent physical morbidity focus on single/few conditions, so cannot investigate the degree to which results reflect a generalised pattern.

**What this study adds?**

- We observed a predominant pattern of an emerging/increasing female ‘excess’ in a broad range of physical morbidity measures between ages 4 and 13 years (rather than no sex differences or an emerging/increasing male ‘excess’).
- Further studies are required to corroborate and explain these findings and understand long-term implications for sex differences in adult health.

## Introduction

An emerging or increasing female ‘excess’ in *psychological* morbidity^1 2^ over the transition to adolescence is well recognised. Evidence of an emerging/increasing female ‘excess’ in several measures of common *physical* morbidity is less well established.^3–5^ Furthermore, since most epidemiological studies focus on single, or small groups of conditions, the degree to which this might reflect a *generalised* pattern in sex differences in physical morbidity has rarely been investigated.

A previous systematic review and meta-analysis, based on a range of self-reported/routinely collected physical morbidity measures in Western children and adolescents, examined whether higher prevalence among males in childhood is replaced by higher prevalence among females in adolescence.^5^ It found an emerging/increasing female ‘excess’ with increasing age for *self-reported* general health and several specific symptoms. This pattern was strongest for headache, abdominal pain and tiredness and weaker for back pain and dizziness. It was also evident for self-reported migraine, but *not* two conditions based on *routinely collected data,* epilepsy (no sex differences) and type 1 diabetes (weak emerging/increasing *male* ‘excess’). The age when a female ‘excess’ was first evident varied by morbidity measure; around 6–8 years for self-assessed health, abdominal pain, dizziness and headache (earlier than expected given previous literature associating female puberty with these physical symptoms^6–8^), and around 9–11 years for back pain, sleeping problems/tiredness and migraine.

More robust evidence for an emerging/increasing female ‘excess’ in self-report than routinely collected data^5^ suggests the need for careful consideration of the impact of data source in studies/reviews focusing on morbidity measured over the transition to adolescence. Changing sex differences with age may reflect underlying biological changes, or societal expectations, of which conditions, symptoms or infections are more ‘appropriate’ for either males or females at different life stages. Apparent sex-by-age differences in morbidity may therefore partly reflect whether data are self-reported (impossible at younger ages), proxy-reported (requiring awareness of symptoms by another, with or without them having been specifically informed by the sufferer) or routinely collected (requiring awareness of symptoms by the sufferer or another, followed by presentation to, and diagnosis by, health professionals).

Further evidence from other reviews broadly suggests an emerging female ‘excess’ occurring around puberty in several measures of common physical morbidity, including asthma,^9–12^ eczema^12 13^ and respiratory infections,^14^ a consistent female excess in both urinary tract infections^15 16^ and musculoskeletal pain,^17^ and no clear sex-by-age pattern in food allergy.^12^ However, many such reviews do not consider the issue of data source,^9 10 14 15 17^ and while others refer to different methods/condition presentations, potential impacts on results are not considered systematically^11 16^ or at all.^12 13^ Similarly, some studies of sex-by-age differences in common childhood/adolescent conditions are unclear about data source,^18^ or based on parental report at younger ages and self-report thereafter, but without acknowledging this might have impacted on results.^19 20^

This paper presents analysis of sex differences in a wide range of *parent/carer-reported* (almost all mother-reported) physical morbidity measures (general health; conditions and symptoms; infections) between ages of 4 and 13 in a large UK birth cohort. This allows exploration of:

1. Whether there is evidence of an emerging/increasing female ‘excess’ *across* these measures, reflecting a generalised pattern;
2. When any emerging female ‘excess’ occurs;
3. Whether the sex-by-age patterns seen in the more restricted set of *child/adolescent self-reported* physical morbidity measures and described in a previous systematic review^5^ are replicated in *parent-reported* measures.

## Method

### Participants

Data are from the Avon Longitudinal Study of Parents and Children (ALSPAC),^21 22^ a population-based cohort study in South-West England. Pregnant women with estimated delivery date 1 April 1991 to 31 December 1992, were invited to participate, resulting in a cohort of 14 541 pregnancies, with 13 988 singletons and first-born twins alive at 1 year.

ALSPAC’s study website includes details of available data via a fully searchable data dictionary (www.bristol.ac.uk/alspac/researchers/access/). All data for this analysis were from ‘child-based’ questionnaires, completed by their main carer at roughly 1 year intervals, from approximately age 4 (57 months; n=4967 male and 5252 female questionnaires; 90% (n=9234) completed by mothers) to 13 years (166 months; n=3682 male and 3678 female questionnaires; 93% (n=6809) completed by mothers).

### Measures

Table 1 details the 32 morbidity measures analysed here and the child ages when they were included. Included measures comprised: three general health (health in past month and year, and health-related school absences); 19 conditions and symptoms (diarrhoea, vomiting, cough, high temperature, earache, ear discharge, stomach-ache, rash, wheezing, breathlessness, headache, constipation, lice/scabies, eczema, asthma, hay fever, pains in arms/legs, food/drink allergies, other allergies) and 10 infections (chicken pox, cold sores, eye infection, ear infection, chest infection, tonsillitis/laryngitis, influenza, cold, urinary infection, worm infections).

**Table 1:** Morbidity measures and ages collected

Measure	How coded for analysis/additional notes	Age in months									
		57	65	69	81	91	103	128	140	157	166
General health											
How would you assess the health of your child in the past month? In the past year?	‘Sometimes quite ill’ or ‘almost always unwell’ (categorised here together as ‘poor’) vs ‘very healthy, no problems’ or ‘healthy, but a few minor problems’	X		X	X	X	X	X	X	X	X
How many days the child had taken off school in the past year.	For a range of health reasons (infections, asthma/eczema/hay fever, hospital or other investigations/admissions, other reasons). Any days off for any health reason vs none					X	X	X	X		X
Conditions and symptoms											
Has child had any of the following?	Since age 3 (at 57 months); in the past 15 months (at 69 months); in the past year (all other ages)										
Diarrhoea		X		X	X	X	X	X		X	X
Vomiting		X		X	X	X	X	X		X	X
Cough		X		X	X	X	X	X		X	X
High temperature		X		X	X	X	X	X		X	X
Earache		X		X	X	X	X	X		X	X
Ear discharge (pus)		X		X	X	X	X	X		X	X
Stomach-ache(s)		X		X	X	X	X	X		X	X
Rash		X		X	X	X	X	X		X	X
Wheezing		X		X	X	X	X	X		X	X
Breathlessness		X		X	X	X	X	X		X	X
Headache(s)		X		X	X	X	X	X		X	X
Constipation		X		X	X	X	X	X		X	X
Lice or scabies	Asked separately from 81 months; combined for consistency in analyses	X		X	X	X	X	X		X	X
Eczema					X	X	X	X		X	X
Asthma					X	X	X	X		X	X
Hay fever					X	X	X	X		X	X
Child often has pains in arms or legs		X		X	X		X		X	X	
Are there any foods or drinks that your child is allergic to?			X		X		X			X	
Apart from food and drink are there any other things to which child is allergic?			X		X		X			X	
Snuffles/cold*		X		X	X	X	X				
Urinary infection†		X		X	X	X	X				
Blood in stools‡		X		X	X	X	X	X		X	X
Convulsions/fits‡		X		X	X	X	X	X		X	X
Episodes of stopping breathing‡		X		X	X	X	X	X		X	X
Convulsion, fit or seizure due to epilepsy‡		X		X	X		X		X		
Accident§		X		X	X	X	X	X		X	X
Infections											
Has child had any of the following infections?	Since age 3 (at 57 months); in the past 15 months (at 69 months); in the past year (all other ages)										
Chicken pox		X		X	X	X	X	X			X
Cold sores		X		X	X	X	X	X			X
Eye infection		X		X	X	X	X	X			X
Ear infection		X		X	X	X	X	X			X
Chest infection		X		X	X	X	X	X			X
Tonsilitis/laryngitis						X	X	X			X
Influenza						X	X	X			X
Cold*						X	X	X			X
Urinary infection†		X		X	X	X	X	X			X
Worm infections¶		X		X	X	X	X	X		X	X
Measles‡		X		X	X	X	X	X			X
Mumps‡		X		X	X	X	X	X			X
Meningitis‡		X		X	X	X	X	X			X
Whooping cough‡		X		X	X	X	X	X			X
German measles‡						X	X	X			X
Scarlet fever‡						X	X	X			X
Glandular fever**											X

*Reported by parent as ‘cold/snuffles’ (within conditions list) at 57, 69, 81, 91 and 103 months and ‘cold’ (within infections list) at 128, 157 and 166 months were combined and included here as ‘cold’ (categorised under infections).

†Reported by parent within both conditions (57–103 months) and infections (57–166 months) lists, so only results in respect of infections data included here.

‡Excluded from analyses as reported in respect of very small numbers (fewer than 20 males and/or females).

§Excluded from analyses as out with the scope of the paper.

¶Reported by parent within conditions list, but included here within infections.

**Excluded from analysis as only one time point.

### Analyses

For each morbidity measure, age-specific logistic regression analyses determined the odds of it being reported in respect of females (vs males). Sex-by-age interactions (testing for a changing sex difference with age) were then included in further logistic regression analyses, based on reports at all ages, with robust SEs to allow for non-independence of observations from the same child.

Results are presented as graphs for each measure, showing female versus male ORs at each age. The graphs have logarithmic scaling (eg, ORs of 2 (females twice as likely) and 0.5 (females half as likely) are the same distance from 1 (no sex difference)). The graphs are presented in three sections (general health; conditions and symptoms; infections) and, within these, according to potential patterns of sex-by-age differences, which a previous systematic review conceptualised in terms of four ‘types’,^5^ and are defined here as:

- ‘Type 1’: an emerging/increasing female ‘excess’, or disappearing male excess with age, occurring because female rates increase more than those of males or decrease less than those of males, resulting in a marked sex-by-age interaction. Type 1 patterns therefore include: (a) a male ‘excess’ reversing to a female ‘excess’, or no sex difference at younger ages, but a female ‘excess’ at older ages (emerging female ‘excess’); (b) a female ‘excess’ at younger ages, increasing with age (increasing female ‘excess’) or (c) a male ‘excess’ at younger ages, but no sex difference at older ages (disappearing male ‘excess’). For ‘type 1’ patterns, the odds of morbidity among females compared with males start below, at, or above unity and increase with age.
- ‘Type 2’: (a) stable female ‘excess’; (b) stable lack of a sex difference or (c) stable male ‘excess’. For ‘type 2’ patterns, the odds of female versus male morbidity are consistently either above, below or at unity.
- ‘Type 3’: variations on an emerging/increasing *male* ‘excess’, or disappearing female ‘excess’ (the reverse of ‘type 1’). For ‘type 3’ patterns, the odds of female versus male morbidity start above, at or below unity and decrease with age.
- ‘Type 4’: mixed/unclassifiable patterns.

(Note precise definitions are included as footnotes to the results graphs.)

Results are based on cross-sectional samples at each age. Two sets of sensitivity analyses were completed. One was restricted to a longitudinal subsample, selecting only those for whom data were available at all relevant ages, n=4454. The other was conducted for the ‘mother-only’ subsample, selecting only mother-completed data, to investigate whether completion by different carers at different ages might impact on the results. Online supplementary table 1 shows cross-sectional, longitudinal and mother-only sample sizes. Online supplementary table 2 shows the characteristics of those included/not included in the samples and demonstrates that due to sample attrition, those included were more likely to be first-born children of mothers in a first marriage, of higher socioeconomic status, and who had never smoked. Results from both the longitudinal and mother-only samples were almost identical to the cross-sectional results. Online supplementary tables 3–5 show results based on each sample. Finally, given that some have suggested Poisson as an alternative to logistic regression for analysis of cross-sectional studies with binary outcomes,^23^ we also conducted Poisson regression analyses on the cross-sectional samples. Results (expressed as risk ratios, rather than ORs) were very similar to those obtained via logistic regression (see online supplementary tables 6–8).

10.1136/bmjpo-2017-000191.supp1 Supplementary file 1

## Results

### General health

**Figure 1:**
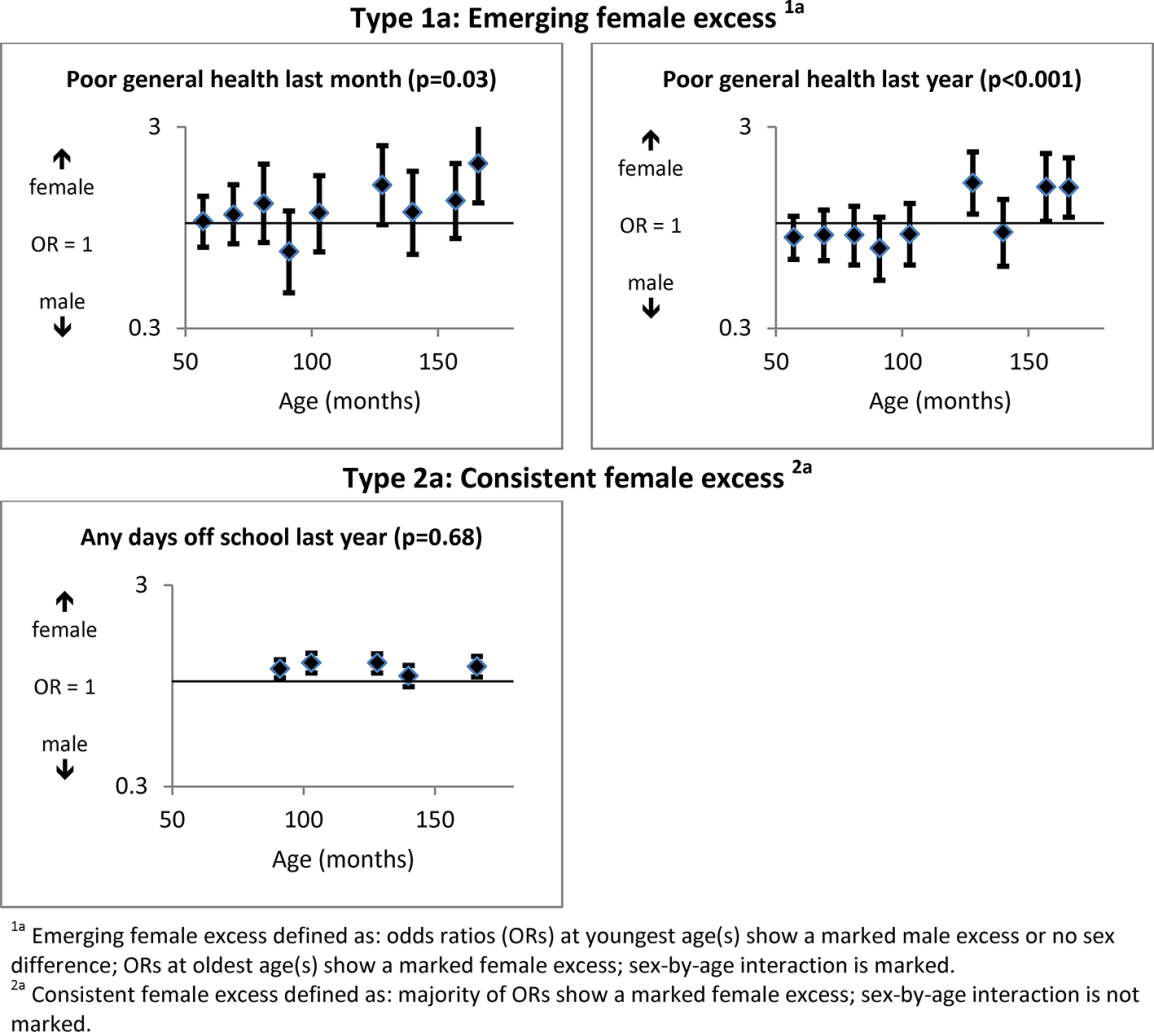
General health measures (with P values for significance of sex-by-age interactions).

Figure 1 shows a ‘type 1a’ female ‘excess’ emerging by 128 months for parent-reported poor general health in their child over the last month (OR=1.02, 95% CI 0.76 to 1.36 at 57 months; OR=1.97, 95% CI 1.26 to 3.10 at 166 months; sex-by-age interaction P=0.01) and the last year (OR=0.85, 95% CI 0.66 to 1.08 at 57 months; OR=1.50, 95% CI 1.07 to 2.11 at 166 months; interaction P<0.001). Days off school in the last year was unavailable at the youngest ages, but showed a small consistent female ‘excess’ (‘type 2a’ pattern) from 91 to 166 months (OR=1.15, 95% CI 1.04 to 1.28 at 91 months; OR=1.18, 95% CI 1.05 to 1.33 at 166 months; interaction P=0.68).

### Conditions and symptoms

**Figure 2:**
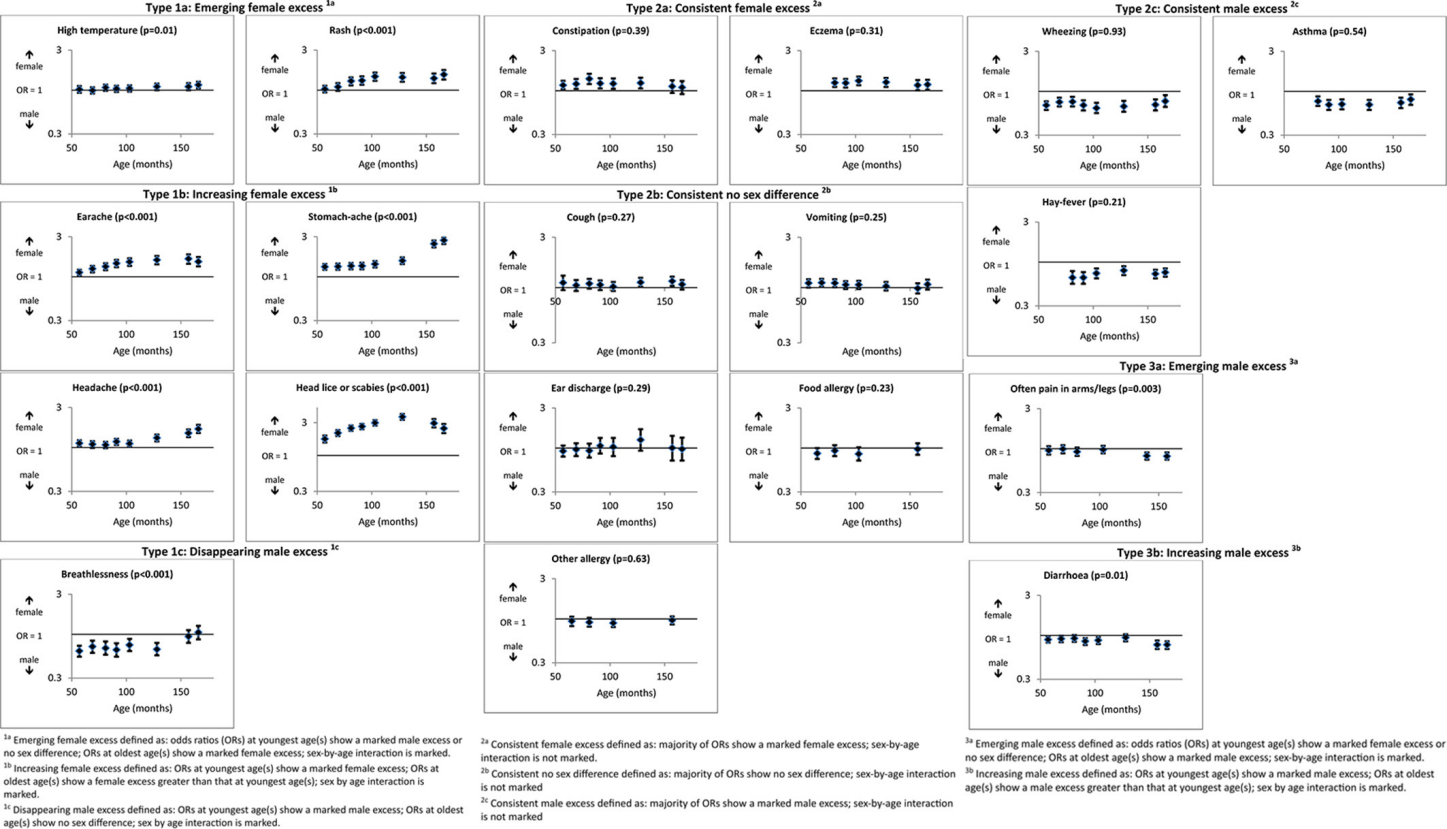
Conditions/symptoms measures (with P values for significance of sex-by-age interactions).

Figure 2 shows that for 6 of the 20 conditions/symptoms measures, there was an emerging/increasing female ‘excess’, while one showed a disappearing male ‘excess’ (all ‘type 1’ patterns). Thus, although there were no sex differences at younger ages, a female ‘excess’ emerged in respect of rates of parent-reported high temperature by 128 months and, more markedly, rash by 81 months (interaction P=0.01 and <0.001, respectively). Earache, stomach-ache, headache and head lice/scabies were more likely to be reported in respect of females at younger ages, but this female ‘excess’ increased with age (interaction P<0.001). Reported breathlessness also showed a type 1 sex-by-age interaction, from a male ‘excess’ at younger ages which disappeared, resulting in no sex difference at older ages (OR=0.63, 95% CI 0.54 to 0.73 at 57 months; OR=1.05, 95% CI 0.87 to 1.26 at 166 months; interaction P<0.001).

Ten conditions/symptoms showed stable (‘type 2’) sex differences/lack of sex differences with age. Thus, two (constipation and eczema) showed a female ‘excess’ at almost all ages and three a consistent male ‘excess’ (wheezing, asthma and hay fever). A further five were largely consistent in showing no marked sex difference at any age (cough, vomiting, ear discharge, food and other allergy).

Finally, two conditions/symptoms (pain in arms/legs and diarrhoea) showed an emerging/increasing male ‘excess’ (‘type 3’ patterns). There was no sex difference in respect of pain in arms/legs at younger ages (OR=0.95, 95% CI 0.85 to 1.07 at 57 months), but a small male ‘excess’ emerged by 140 months and was maintained at 157 months (OR=0.81, 95% CI 0.74 to 0.90 at 157 months; interaction P=0.001). Diarrhoea showed an increasing male ‘excess’, being more likely to be reported in males at all ages, particularly older ages (OR=0.89, 95% CI 0.82 to 0.96 at 57 months; OR=0.77, 95% CI 0.69 to 0.85 at 166 months; interaction P=0.004).

### Infections

**Figure 3:**
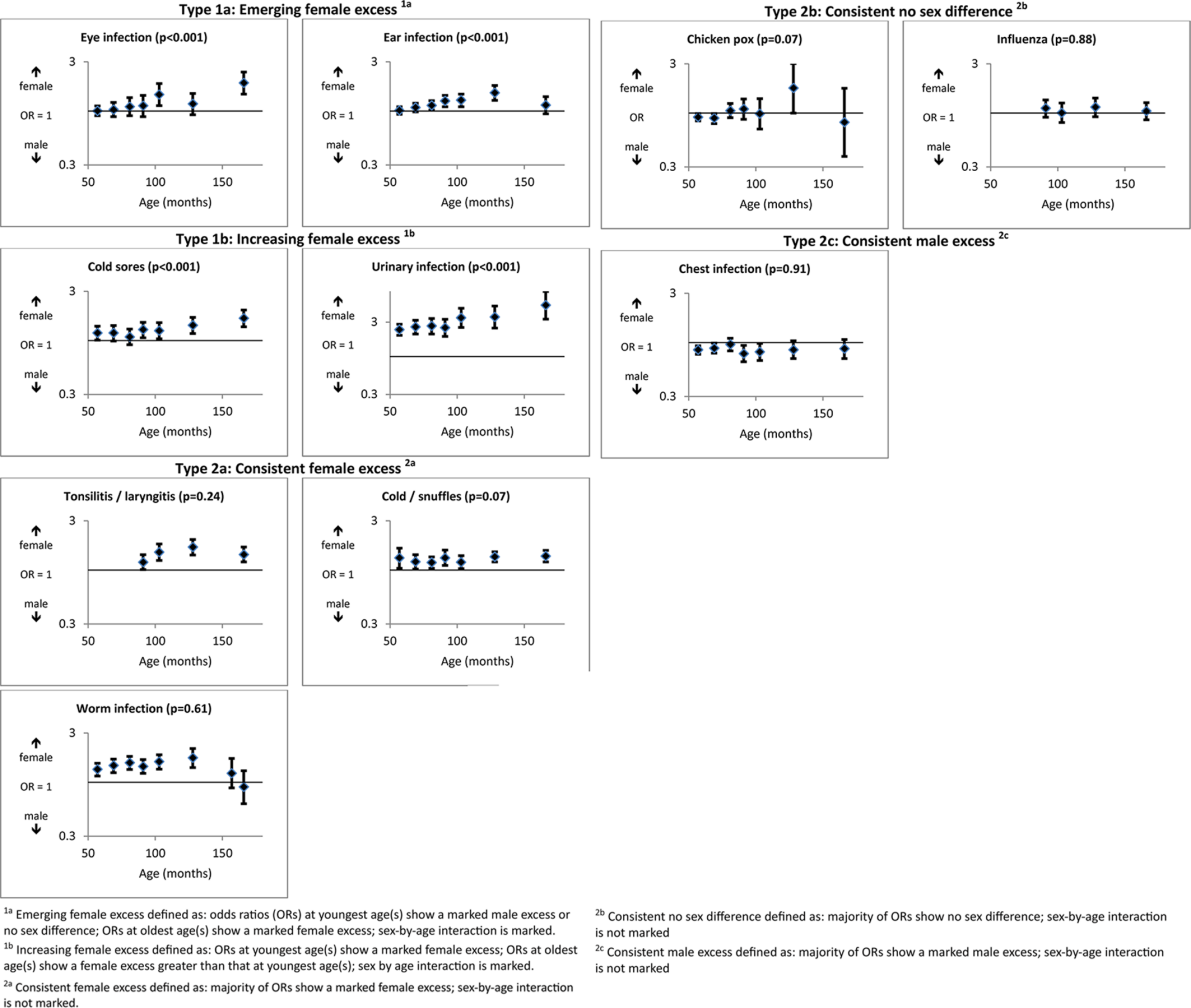
Infections measures (with P values for significance of sex-by-age interactions).

Figure 3 shows an emerging/increasing female ‘excess’ in 4 of the 11 parent-reported infections (‘type 1’ patterns), a consistent female ‘excess’ in 3, a consistent male ‘excess’ in 1 and no sex difference in 2 (‘type 2’ patterns).

Among infections showing an emerging/increasing female ‘excess’, there were no sex differences in parent-reported child eye and ear infections at younger ages, but a female ‘excess’ emerged for eye infections by 103 months and ear infections by 81 months (interaction P<0.001 for both). Parent-reported cold sores and, more markedly, urinary infections (note different scale on graph) were higher in females0 at all ages, but this sex difference increased with age (eg, for urinary infections, OR=2.35, 95% CI 1.97 to 2.81 at 57 months; OR=5.13, 95% CI 3.30 to 7.98 at 166 months; interaction P<0.001). Finally, among the infections showing ‘type 2’ patterns, there was a consistent female ‘excess’ in respect of reported tonsillitis, cold/snuffles and worm infections, no sex difference in chicken pox and influenza and a consistent male ‘excess’ in respect of chest infections.

## Discussion

This is the first study to examine sex differences in a range of parent-reported physical morbidity measures during childhood and adolescence and explore age-based changes.(Table 2)

**Table 2:** Summary of patterns of sex-by-age differences in each morbidity measure

	General health	Conditions and symptoms	Infections
‘Type 1’			
1a: Emerging female excess	General health – Past year General health – Past month	High temperature Rash	Eye infection Ear infection
1b: Increasing female excess		Earache Stomach-ache Headache Lice/scabies	Cold sores Urinary infection
1c: Disappearing male excess		Breathlessness	
‘Type 2’			
2a: Consistent female excess	Health-related days off school	Constipation Eczema	Tonsillitis/laryngitis Cold Worm infections
2b: Consistent no sex difference		Vomiting Cough Ear discharge Food/drink allergies Non-food/drink allergies	Chicken pox Influenza
2c: Consistent male excess		Wheezing Asthma Hay fever	Chest infection
‘Type 3’			
3a: Emerging male excess		Pain in arms/legs	
3b: Increasing male excess		Diarrhoea	

Given evidence of a generalised pattern for *psychological* morbidity measures, we were interested in whether there was also a generalised pattern of an emerging/increasing female ‘excess’ across these *physical morbidity measures* and, if so, when this occurred. As summarised in table 2, of the 32 measures examined, only 7 showed *no sex differences* throughout the included age ranges. Six were categorised as showing an *emerging female ‘excess’*, six an *increasing female ‘excess’* one a *disappearing male ‘excess’* (‘type 1’ patterns) and six a *consistent female ‘excess’*. In contrast, only one showed an *emerging male ‘excess’,* one an *increasing male ‘excess’* (‘type 3’ patterns) and four a *consistent male ‘excess’*. Thus, far more measures showed an emerging/increasing female ‘excess’ than an emerging/increasing male ‘excess’ or no sex difference.

We also wished to know whether sex-by-age patterns in a more restricted set of *child/adolescent self-reported* physical morbidity measures described in a previous review,^5^ were replicated when these measures were *parent-reported,* as here. Three measures (general health, headache, abdominal pain) are included in both studies. The review found a marked female ‘excess’ in each, based on self-reports, from around 6–8 years. The current analysis, based on parent-reported measures, had broadly similar findings, but suggested an even earlier small female ‘excess’ in headache and stomach-ache.

How can these complex patterns of sex differences in parent-reported morbidity be explained? Female puberty is associated with physical symptoms, including menstrual cramps and headaches.^6–8^ Although previous literature suggests higher male rates of asthma, eczema, respiratory infections and perhaps hay fever at younger ages, reversing around puberty,^9–14 18 19^ we observed a consistent male ‘excess’ in asthma, wheeze, hay fever and chest infections throughout the age range considered here. It is possible that a ‘reversal’ occurred later in puberty in the ALSPAC cohort. However, the consistently higher female eczema rates cannot be explained in this way. While potential explanations might be constructed for some findings (eg, the increasing female ‘excess’ in lice/scabies throughout childhood might result from girls’ often longer hair and/or greater time spent in physically close social interactions^24^), others, such as an emerging/increasing female ‘excesses’ in temperature, rash, earache/infection, eye infection or cold sores are harder to explain.

Another potential explanation is that some of these sex differences in parent-reported morbidity measures result from different illness-related attitudes/expectations (by both children and parents) in respect of males compared with females. Parental expectations about, and reinforcement of, their child’s emotional expressivity differ according to child sex,^25^ as do ratings of, and responses to, paediatric pain.^26 27^ Perhaps these translate into differences in acknowledging, recalling and reporting illness in respect of boys and girls. There are few studies in this area and stereotyped attitudes and expectations relating to sex differences may differ according to what aspect of child morbidity is being considered.

The strengths of this study include its large sample size and wide range of morbidity measures, enabling us to address previously unexplored questions. Analyses based on cross-sectional samples produced almost identical results to those limited to the longitudinal sample (which was subject to differential attrition) and mother-only samples (which eliminated different carers reporting on a child’s health at different ages). Findings such as the much higher rates of urinary infections among females at all ages and markedly increasing female ‘excess’ in stomach and headaches in early adolescence, are consistent with other research.^6–8 15 16^ However, we also draw attention to potential limitations. Our categorisation of patterns of sex-by-age differences in physical morbidity, based on graphs and interactions, could be regarded as simple, and the fact that we conducted analyses on 32 morbidity measures introduces the possibility of spurious (chance) significance for some sex-by-age interactions due to multiple testing. However, our focus was on consistent patterns of ORs with age and these are unlikely to have arisen purely by chance. In addition, there is no reason to think that spurious interactions would occur more often for measures showing a ‘type 1’ pattern. Another limitation is that this secondary analysis of existing data from a well-established cohort was inevitably limited by the specific measures chosen by the ALSPAC team at each age. In particular, lack of comparable data at older ages prevents extension of the analysis to mid-later adolescence. Furthermore, although there was little evidence of differential attrition according to child sex, the sample was more advantaged than the general population, thus potentially limiting generalisability of the findings.

Based on rigorous analysis questioning, we believe for the first time, whether age-based changes in sex differences in child and adolescent physical morbidity follow specific patterns (as proposed in a prior review^5^), this analysis suggests both substantive and methodological conclusions. Substantively, it is intriguing that sex differences are evident in respect of a wide range of parent-reported physical morbidity measures in childhood and early adolescence, generally indicating poorer health in girls. While some measures show consistent female or male ‘excesses’, many show an emerging/increasing female ‘excess’ in childhood, consistent with findings for psychological morbidity,^1 2^ which are evident prepuberty; almost none shows an emerging/increasing male ‘excess’. This pattern of ‘excess’ female morbidity by/before puberty highlights important inequities, with public health implications, not least in future health service usage. Many of these (changing) sex differences are hard to explain on the basis of existing literature, suggesting, as in adults,^28^ the need for further quantitative studies to corroborate these findings and to examine whether patterns differ across sociodemographic or cultural groups. There is also a need for further qualitative or experimental studies to examine the social and/or biological mechanisms underlying these findings.^29 30^ Methodologically, possible differences according to data source (routine data, child/proxy-reported) highlight the need for reviews of sex-by-age differences in child/adolescent morbidity to pay close attention to this issue and for studies which systematically compare results based on multiple sources.
