# Supplementary material for: Sex differences in child and adolescent physical morbidity: cohort study
Source: BMJ Paediatr Open. 2017 Dec 29;1(1):e000191. doi: 10.1136/bmjpo-2017-000191 (PMC5862201; doi:10.1136/bmjpo-2017-000191)
Supplement: Supplementary file 1 [file bmjpo-2017-000191supp001.pdf]

Supplementary Table 1: Sample sizes at each age*

	Cross-sectional samples			Longitudinal sample			Mother-only cross-sectional samples		
	N (%) male	N (%) female	Total	N (%) male	N (%) female	Total	N (%) male	N (%) female	Total
57 months	5,252 (51.4)	4,967 (48.6)	10,219	2,254 (50.6)	2,200 (49.4)	4,454	4,769 (51.7)	4,465 (48.4)	9,234
65 months	4,956 (51.1)	4,749 (48.9)	9,705	2,254 (50.6)	2,200 (49.4)	4,454	4,443 (51.3)	4,213 (48.7)	8,656
69 months	4,799 (51.1)	4,598 (48.9)	9,397	2,254 (50.6)	2,200 (49.4)	4,454	4,300 (51.3)	4,082 (48.7)	8,382
81 months	4,374 (51.5)	4,128 (48.6)	8,502	2,254 (50.6)	2,200 (49.4)	4,454	4,236 (51.4)	4,010 (48.6)	8,246
91 months	4,245 (51.4)	4,011 (48.6)	8,256	2,254 (50.6)	2,200 (49.4)	4,454	4,123 (51.3)	3,917 (48.7)	8,040
103 months	4,348 (50.7)	4,234 (49.3)	8,582	2,254 (50.6)	2,200 (49.4)	4,454	4,081 (51.0)	3,923 (49.0)	8,004
128 months	4,059 (50.2)	4,028 (49.8)	8,087	2,254 (50.6)	2,200 (49.4)	4,454	3,804 (50.1)	3,788 (49.9)	7,592
140 months	3,842 (49.8)	3,874 (50.2)	7,716	2,254 (50.6)	2,200 (49.4)	4,454	3,618 (49.9)	3,633 (50.1)	7,251
157 months	3.674 (49.7)	3,724 (50.3)	7,398	2,254 (50.6)	2,200 (49.4)	4,454	3,437 (49.7)	3,477 (50.3)	6,914
166 months	3,678 (50.0)	3,682 (50.0)	7,360	2,254 (50.6)	2,200 (49.4)	4,454	3,388 (49.8)	3,421 (50.2)	6,809

*** Note maximum sample size at each age – individual missing values will further reduce sample sizes.**

Supplementary Table 2: Characteristics of those included/not included in analytical samples (column percentages)

	First cross-sectional sample – 57 month questionnaire		Final cross-sectional sample – 166 month questionnaire		Longitudinal sample – all questionnaires	
	Completed (N=10,291)	Not completed (N=5,154)	Completed (N=7,360)	Not completed (N=8,085)	All completed (N=4,454)	At least one not completed (N=10,991)
**Child sex**						
Male	51.4	51.4	50.0	52.8	50.6	51.8
Female	48.6	48.6	50.0	47.2	49.4	48.3
**Child birth order**						
First-born	45.9	42.1	47.6	41.8	48.6	42.8
Second-born	35.3	34.2	35.4	35.5	35.4	34.7
Later	18.8	23.7	17.0	22.7	16.0	22.5
**Maternal marital status during pregnancy**						
Never married	15.7	26.8	13.7	24.5	10.8	23.2
In first marriage	72.3	59.3	74.8	62.0	78.4	63.4
Other	12.0	13.9	11.5	13.5	10.8	13.4
**Maternal housing tenure during pregnancy**						
Owner occupant	79.3	59.8	82.9	63.7	86.8	66.7
Renting	20.7	40.2	17.1	36.3	13.2	33.3
**Maternal education during pregnancy**						
O level or more	75.0	55.6	79.2	59.4	82.4	63.1
Less	25.0	44.4	20.8	40.6	17.6	36.9
**Maternal smoking during pregnancy**						
Ever	47.5	58.9	44.0	57.9	40.9	55.8
Never	52.5	41.1	56.0	42.1	59.1	44.2

Supplementary Table 3: OR (95% CI) for general health measures in females compared with males

	Cross-sectional samples		Longitudinal sample		Mother-only cross-sectional samples	
	**N (%) outcome females / males**	**OR (95% CI)**	**N (%) outcome females / males**	**OR (95% CI)**	**N (%) outcome females / males**	**OR (95% CI)**
**Poor general health last month**						
57 months	89 (2.0)/94 (2.0)	1.02 (0.76, 1.36)	24 (1.3)/30 (1.6)	0.80 (0.46, 1.37)	89 (2.1)/90 (2.0)	1.05 (0.78, 1.42)
69 months	69 (1.7)/67 (1.5)	1.10 (0.79, 1.55)	27 (1.5)/28 (1.5)	0.96 (0.56, 1.64)	68 (1.7)/65 (1.5)	1.11 (0.79, 1.57)
81 months	42 (1.1)/35 (0.9)	1.25 (0.80, 1.96)	16 (0.9)/17 (0.9)	0.94 (0.47, 1.86)	42 (1.1)/34 (0.9)	1.29 (0.82, 2.03)
91 months	30 (0.8)/44 (1.0)	0.72 (0.45, 1.15)	10 (0.5)/21 (1.1)	0.47 (0.22, 1.01)	30 (0.8)/42 (1.0)	0.75 (0.47, 1.20)
103 months	43 (1.1)/40 (1.0)	1.12 (0.72, 1.72)	10 (0.5)/15 (0.8)	0.66 (0.30, 1.48)	42 (1.1)/39 (1.0)	1.12 (0.72, 1.73)
128 months	48 (1.3)/32 (0.8)	1.54 (0.98, 2.42)	22 (1.2)/17 (0.9)	1.30 (0.69, 2.45)	47 (1.3)/30 (0.8)	1.59 (1.00, 2.51)
140 months	37 (1.0)/33 (0.9)	1.13 (0.70, 1.81)	14 (0.8)/15 (0.8)	0.93 (0.45, 1.93)	35 (1.0)/33 (0.9)	1.06 (0.66, 1.71)
157 months	49 (1.4)/38 (1.1)	1.29 (0.84, 1.98)	18 (1.0)/20 (1.1)	0.90 (0.47, 1.70)	49 (1.4)/37 (1.1)	1.32 (0.86, 2.03)
166 months	56 (1.6)/29 (0.8)	1.97 (1.26, 3.10)	26 (1.4)/14 (0.8)	1.87 (0.97, 3.59)	55 (1.6)/28 (0.8)	1.97 (1.25, 3.12)
**F:M OR (95% CI) per year of age**		**1.06 (1.01, 1.12)**		**1.07 (0.98, 1.16)**		**1.06 (1.00, 1.11)**
P(interaction) with sex		***0.03***		***0.12***		***0.04***
**Poor general health in last year**						
57 months	120 (2.7)/150 (3.2)	0.85 (0.66, 1.08)	25 (1.5)/40 (2.4)	0.62 (0.37, 1.02)	118 (2.7)/145 (3.2)	0.85 (0.67, 1.09)
69 months	85 (2.1)/103 (2.4)	0.87 (0.65, 1.16)	27 (1.6)/36 (2.2)	0.74 (0.45, 1.23)	83 (2.1)/100 (2.4)	0.87 (0.65, 1.17)
81 months	65 (1.7)/78 (2.0)	0.87 (0.62, 1.21)	22 (1.3)/25 (1.5)	0.87 (0.49, 1.55)	65 (1.7)/77 (2.0)	0.87 (0.63, 1.22)
91 months	51 (1.3)/72 (1.7)	0.75 (0.52, 1.07)	14 (0.8)/23 (1.4)	0.60 (0.31, 1.17)	50 (1.3)/70 (1.7)	0.75 (0.52, 1.08)
103 months	60 (1.5)/70 (1.7)	0.88 (0.62, 1.25)	10 (0.6)/19 (1.1)	0.52 (0.24, 1.12)	58 (1.5)/67 (1.7)	0.89 (0.62, 1.27)
128 months	78 (2.1)/51 (1.4)	1.58 (1.11, 2.26)	27 (1.6)/20 (1.2)	1.35 (0.75, 2.41)	75 (2.1)/49 (1.3)	1.56 (1.09, 2.24)
140 months	51 (1.4)/57 (1.6)	0.90 (0.61, 1.31)	17 (1.0)/21 (1.3)	0.80 (0.42, 1.53)	49 (1.4)/56 (1.6)	0.87 (0.59, 1.28)
157 months	65 (1.9)/43 (1.3)	1.51 (1.02, 2.22)	24 (1.4)/19 (1.1)	1.26 (0.69, 2.31)	63 (1.9)/41 (1.3)	1.52 (1.02, 2.26)
166 months	83 (2.5)/57 (1.7)	1.50 (1.07, 2.11)	32 (1.9)/27 (1.6)	1.18 (0.70, 1.98)	79 (2.4)/54 (1.7)	1.48 (1.04, 2.10)
**F:M OR (95% CI) per year of age**		**1.08 (1.03, 1.13)**		**1.09 (1.00, 1.17)**		**1.08 (1.03, 1.12)**
P(interaction) with sex		***0.001***		***0.04***		***0.001***
**Any days off school in last year**						
91 months	3,067 (77.6)/3,142 (75.0)	1.15 (1.04, 1.28)	1,808 (77.7)/1,790 (76.2)	1.09 (0.95, 1.25)	3,016 (77.7)/3,079 (75.4)	1.14 (1.03, 1.26)
103 months	3,264 (82.5)/3,238 (79.3)	1.23 (1.10, 1.38)	1,904 (81.9)/1,842 (78.4)	1.24 (1.08, 1.43)	3,179 (82.6)/3,156 (79.5)	1.22 (1.09, 1.37)
128 months	3,078 (80.5)/3,008 (77.0)	1.23 (1.10, 1.37)	1,850 (79.5)/1,839 (78.3)	1.08 (0.94, 1.24)	3,037 (80.7)/2,925 (77.2)	1.23 (1.10, 1.38)
140 months	3,015 (83.5)/2,984 (82.7)	1.06 (0.94, 1.20)	1,923 (82.7)/1,917 (81.6)	1.08 (0.93, 1.25)	2,964 (83.4)/2,926 (82.8)	1.05 (0.93, 1.19)
166 months	2,841 (81.8)/2,779 (79.2)	1.18 (1.05, 1.33)	1,925 (82.8)/1,860 (79.2)	1.26 (1.09, 1.46)	2,781 (82.0)/2,673 (79.4)	1.18 (1.05, 1.34)
**F:M OR (95% CI) per year of age**		**1.00 (0.97, 1.02)**		**1.01 (0.98, 1.04)**		**1.00 (0.97, 1.02)**
P(interaction) with sex		***0.68***		***0.49***		***0.81***

Supplementary Table 4: OR (95% CI) for conditions in females compared with males

	Cross-sectional samples		Longitudinal sample		Mother-only cross-sectional samples	
	N (%) outcome females / males	OR (95% CI)	N (%) outcome females / males	OR (95% CI)	N (%) outcome females / males	OR (95% CI)
**Diarrhoea**						
57 months	2,634 (58.1)/2,967 (61.0)	0.89 (0.82, 0.96)	1,173 (57.0)/1,249 (60.5)	0.86 (0.76, 0.98)	2,593 (58.3)/2,890 (61.1)	0.89 (0.82, 0.97)
69 months	1,592 (39.3)/1,796 (41.6)	0.91 (0.83, 099)	776 (37.7)/873 (42.3)	0.83 (0.73, 0.93)	1,561 (39.4)/1,750 (41.7)	0.91 (0.83, 0.99)
81 months	1,422 (34.5)/1,586 (36.3)	0.92 (0.84, 1.01)	682 (33.1)/723 (35.0)	0.92 (0.81, 1.04)	1,397 (34.6)/1,550 (36.3)	0.93 (0.85, 1.01)
91 months	1,444 (35.3)/1,228 (31.7)	0.85 (0.77-0.93)	639 (31.0)/720 (34.9)	0.84 (0.74, 0.96)	1,208 (31.8)/1,409 (35.3)	0.85 (0.77, 0.94)
103 months	1,214 (30.7)/1,386 (33.8)	0.87 (0.79, 0.95)	625 (30.4)/655 (31.7)	0.94 (0.82, 1.07)	1,187 (30.8)/1,358 (34.0)	0.87 (0.79, 0.95)
128 months	1,223 (31.7)/1,301 (33.2)	0.94 (0.85, 1.03)	617 (30.0)/654 (31.7)	0.92 (0.81, 1.05)	1,206 (31.8)/1,261 (33.1)	0.94 (0.86, 1.04)
157 months	730 (20.9)/887 (25.4)	0.77 (0.69, 0.87)	415 (20.2)/507 (24.6)	0.78 (0.67, 0.90)	713 (20.9)/869 (25.6)	0.77 (0.68, 0.86)
166 months	908 (26.0)/1,111 (31.5)	0.77 (0.69, 0.85)	500 (24.3)/632 (30.6)	0.73 (0.63, 0.83)	888 (26.0)/1,067 (31.4)	0.77 (0.69, 0.85)
**F:M OR (95% CI) per year of age**		**0.98 (0.97, 1.00)**		**0.98 (0.97, 1.00)**		**0.98 (0.97, 1.00)**
P(interaction) with sex		***0.01***		***0.05***		***0.01***
**Vomiting**						
57 months	3,255 (71.9)/3,413 (70.0)	1.10 (1.00, 1.20)	1,508 (73.5)/1,492 (72.0)	1.08 (0.94, 1.24)	3,205 (72.2)/3,327 (70.2)	1.10 (1.01, 1.20)
69 months	2,098 (51.7)/2,124 (49.1)	1.11 (1.02, 1.21)	1,076 (52.5)/1,048 (50.6)	1.08 (0.96, 1.22)	2,062 (52.0)/2,064 (49.1)	1.12 (1.03, 1.22)
81 months	1,897 (45.6)/1,907 (43.6)	1.10 (1.01, 1.20)	961 (46.9)/948 (45.7)	1.05 (0.93, 1.18)	1,863 (46.1)/1,867 (43.8)	1.10 (1.01, 1.20)
91 months	1,691 (43.5)/1,722 (42.2)	1.06 (0.97, 1.15)	908 (44.3)/869 (41.9)	1.10 (0.97, 1.25)	1,656 (43.4)/1,681 (42.2)	1.05 (0.96, 1.15)
103 months	1,605 (40.5)/1,601 (39.1)	1.06 (0.97, 1.16)	850 (41.4)/815 (39.3)	1.09 (0.96, 1.24)	1,569 (40.7)/1,565 (39.3)	1.06 (0.97, 1.16)
128 months	1,439 (37.5)/1,441 (36.9)	1.03 (0.94, 1.13)	771 (37.6)/772 (37.2)	1.02 (0.89, 1.15)	1,420 (37.6)/1,402 (37.0)	1.03 (0.94, 1.13)
157 months	876 (25.0)/884 (25.3)	0.98 (0.88, 1.10)	497 (24.2)/525 (25.3)	0.94 (0.82, 1.09)	856 (25.0)/858 (25.3)	0.99 (0.88, 1.10)
166 months	996 (28.5)/960 (27.2)	1.07 (0.96, 1.19)	557 (27.2)/569 (27.5)	0.99 (0.86, 1.13)	973 (28.5)/935 (27.6)	1.05 (0.94, 1.16)
**F:M OR (95% CI) per year of age**		**0.99 (0.98, 1.00)**		**0.99 (0.97, 1.00)**		**0.99 (0.98, 1.00)**
P(interaction) with sex		***0.25***		***0.10***		***0.16***
**Cough**						
57 months	4,248 (93.4)/4,527 (91.8)	1.11 (0.95, 1.30)	1,958 (93.6)/1,963 (93.2)	1.06 (0.83, 1.36)	4,170 (93.5)/4,398 (92.7)	1.12 (0.96, 1.32)
69 months	3,490 (85.5)/3,709 (84.9)	1.05 (0.93, 1.18)	1,824 (87.2)/1,829 (86.8)	1.03 (0.86, 1.23)	3,412 (85.5)/3,594 (84.7)	1.06 (0.94, 1.19)
81 months	3,221 (78.0)/3,346 (76.5)	1.09 (0.98, 1.21)	1,656 (79.1)/1,643 (78.0)	1.07 (0.92, 1.24)	3,156 (78.1)/3,261 (76.4)	1.10 (0.99, 1.22)
91 months	2,961 (75.8)/3,080 (74.8)	1.06 (0.95, 1.17)	1,589 (75.9)/1,608 (76.3)	0.98 (0.85, 1.13)	2,902 (75.7)/3,006 (74.8)	1.05 (0.95, 1.17)
103 months	2,947 (73.6)/3,011 (73.1)	1.02 (0.93, 1.13)	1,544 (73.8)/1,566 (74.3)	0.97 (0.85, 1.12)	2,864 (73.4)/2,927 (73.0)	1.02 (0.92, 1.13)
128 months	2,834 (73.5)/2,790 (71.2)	1.12 (1.02, 1.24)	1,567 (74.9)/1,534 (72.8)	1.11 (0.97, 1.28)	2,787 (73.4)/2,707 (71.2)	1.12 (1.01, 1.24)
157 months	2,380 (67.3)/2,259 (64.1)	1.15 (1.04, 1.27)	1,412 (67.5)/1,374 (65.2)	1.11 (0.97, 1.26)	2,326 (67.3)/2,197 (64.3)	1.15 (1.04, 1.27)
166 months	2,452 (70.0)/2,431 (68.7)	1.07 (0.96, 1.18)	1,463 (69.9)/1,486 (70.5)	0.97 (0.85, 1.11)	2,393 (70.0)/2,332 (68.7)	1.06 (0.96, 1.18)
**F:M OR (95% CI) per year of age**		**1.01 (0.99, 1.02)**		**1.00 (0.99, 1.02)**		**1.01 (0.99, 1.02)**
P(interaction) with sex		***0.27***		***0.82***		***0.38***

Supplementary Table 4 continued

	Cross-sectional samples		Longitudinal sample		Mother-only cross-sectional samples	
	N (%) outcome females / males	OR (95% CI)	N (%) outcome females / males	OR (95% CI)	N (%) outcome females / males	OR (95% CI)
**High temperature**						
57 months	3,401 (75.2)/3,636 (74.8)	1.02 (0.93, 1.12)	1,578 (76.3)/1,616 (77.6)	0.93 (0.81, 1.08)	3,346 (75.4)/3,548 (75.1)	1.02 (0.92, 1.12)
69 months	2,495 (61.4)/2,670 (61.7)	0.99 (0.91, 1.08)	1,284 (62.1)/1,294 (62.1)	1.00 (0.88, 1.13)	2,442 (61.5)/2,585 (61.6)	1.00 (0.91, 1.09)
81 months	2,26 (53.9)/2,285 (52.3)	1.07 (0.98, 1.16)	1,140 (55.2)/1,127 (54.1)	1.04 (0.92, 1.18)	2,188 (54.2)/2,234 (52.4)	1.08 (0.99, 1.17)
91 months	2,006 (51.4)/2,073 (50.5)	1.04 (0.95, 1.13)	1,089 (52.7)/1,083 (52.0)	1.03 (0.91, 1.16)	1,969 (51.4)/2,025 (50.5)	1.04 (0.95, 1.13)
103 months	1,948 (48.9)/1,970 (47.8)	1.05 (0.96, 1.14)	1,004 (48.6)/1,006 (48.3)	1.01 (0.90, 1.14)	1,894 (48.9)/1,916 (47.8)	1.04 (0.96, 1.14)
128 months	1,889 (49.1)/1,832 (46.8)	1.10 (1.00, 1.20)	1,026 (49.6)/996 (47.8)	1.08 (0.95, 1.21)	1,857 (49.0)/1,782 (46.9)	1.09 (0.99, 1.19)
157 months	1,342 (38.1)/1,257 (35.9)	1.10 (1.00, 1.21)	789 (38.2)/757 (36.3)	1.08 (0.95, 1.23)	1,317 (38.3)/1,230 (36.2)	1.10 (0.99, 1.21)
166 months	1,533 (43.8)/1,428 (40.4)	1.15 (1,05, 1.26)	909 (44.0)/831 (39.9)	1.18 (1.05, 1.34)	1,494 (43.7)/1,375 (40.5)	1.14 (1.03, 1.25)
**F:M OR (95% CI) per year of age**		**1.01 (1.00, 1.02)**		**1.02 (1.00, 1.03)**		**1.01 (1.00, 1.02)**
P(interaction) with sex		***0.01***		***0.01***		***0.02***
**Earache**						
57 months	1,916 (42.3)/1,933 (39.6)	1.12 (1.03, 1.21)	915 (41.8)/872 (39.2)	1.11 (0.99, 1.26)	1,884 (42.3)/1,886 (39.8)	1.11 (1.02, 1.21)
69 months	1,529 (36.6)/1,413 (31.9)	1.24 (1.13, 1.35)	795 (36.3)/708 (31.8)	1.22 (1.08, 1.38)	1,499 (36.8)/1,374 (31.9)	1.24 (1.13, 1.36)
81 months	1,273 (30.8)/1,103 (25.2)	1.32 (1.20, 1.45)	682 (31.1)/580 (26.0)	1.28 (1.13, 1.46)	1,247 (30.9)/1,077 (25.2)	1.32 (1.20, 1.46)
91 months	1,165 (29.2)/940 (22.3)	1.44 (1.30, 1.59)	630 (28.8)/469 (21.1)	1.51 (1.32, 1.74)	1,142 (29.2)/914 (22.2)	1.45 (1.31, 1.60)
103 months	1.073 (26.6)/817 (19.5)	1.50 (1.35, 1.66)	570 (26.0)/410 (18.4)	1.56 (1.35, 1.80)	1,043 (26.5)/797 (19.5)	1.49 (1.34, 1.65)
128 months	904 (23.5)/638 (16.2)	1.58 (1.41, 1.77)	501 (22.9)/333 (15.0)	1.69 (1.45, 1.97)	889 (23.4)/620 (16.3)	1.58 (1.41, 1.77)
157 months	673 (19.1)/444 (12.7)	1.63 (1.43, 1.85)	399 (18.2)/269 (12.1)	1.62 (1.37, 1.92)	653 (19.0)/433 (12.8)	1.61 (1.41, 1.83)
166 months	675 (19.2)/483 (13.6)	1.51 (1.33, 1.72)	398 (18.2)/286 (12.8)	1.51 (1.28, 1.78)	654 (19.1)/466 (13.7)	1.48 (1.30, 1.69)
**F:M OR (95% CI) per year of age**		**1.04 (1.03, 1.06)**		**1.04 (1.02, 1.06)**		**1.04 (1.03, 1.06)**
P(interaction) with sex		***<0.001***		***<0.001***		***<0.001***
**Ear discharge**						
57 months	323 (7.2)/375 (7.7)	0.92 (0.79, 1.07)	151 (6.9)/160 (7.2)	0.96 (0.76, 1.21)	320 (7.2)/369 (7.8)	0.92 (0.78, 1.07)
69 months	257 (6.2)/285 (6.4)	0.96 (0.80, 1.14)	135 (6.2)/138 (6.2)	1.00 (0.78,1.27)	255 (6.3)/276 (6.4)	0.98 (0.82, 1.16)
81 months	215 (5.2)/243 (5.6)	0.93 (0.77, 1.13)	108 (4.9)/117 (5.3)	0.94 (0.72, 1.22)	212 (5.3)/241 (5.7)	0.93 (0.77, 1.12)
91 months	170 (4.3)/170 (4.0)	1.06 (0.86, 1.32)	95 (4.3)/74 (3.3)	1.32 (0.97, 1.80)	169 (4.3)/167 (4.1)	1.07 (0.86, 1.33)
103 months	128 (3.2)/129 (3.1)	1.03 (0.80, 1.32)	60 (2.7)/57 (2.6)	1.07 (0.74, 1.55)	127 (3.2)/125 (3.1)	1.06 (0.82, 1.36)
128 months	99 (2.6)/81 (2.1)	1.25 (0.93, 1.68)	52 (2.4)/44 (2.0)	1.21 (0.80, 1.81)	97 (2.6)/77 (2.0)	1.27 (0.94, 1.72)
157 months	67 (1.9)/67 (1.9)	1.00 (0.71, 1.40)	33 (1.5)/44 (2.0)	0.76 (0.48, 1.20)	64 (1.9)/65 (1.9)	0.98 (0.69, 1.38)
166 months	90 (2.3)/83 (2.3)	0.97 (0.71, 1.33)	44 (2.0)/53 (2.4)	0.84 (0.56, 1.26)	78 (2.3)/80 (2.4)	0.97 (0.71, 1.33)
**F:M OR (95% CI) per year of age**		**1.02 (0.98, 1.05)**		**0.99 (0.94, 1.04)**		**1.02 (0.98, 1.05)**
P(interaction) with sex		***0.29***		***0.64***		***0.32***

Supplementary Table 4 continued

	Cross-sectional samples		Longitudinal sample		Mother-only cross-sectional samples	
	N (%) outcome females / males	OR (95% CI)	N (%) outcome females / males	OR (95% CI)	N (%) outcome females / males	OR (95% CI)
**Stomach ache**						
57 months	2,549 (56.2)/2,405 (49.4)	1.31 (1.21, 1.42)	1,236 (56.2)/1,110 (49.6)	1.30 (1.15, 1.46)	2,514 (56.4)/2,342 (49.5)	1.32 (1.22, 1.44)
69 months	2,390 (57.4)/2,243 (50.5)	1.32 (1.21, 1.44)	1,263 (57.4)/1,158 (51.8)	1.25 (1.11, 1.41)	2,335 (57.3)/2,181 (50.6)	1.31 (1.21, 1.43)
81 months	2,607 (63.2)/2,453 (56.1)	1.34 (1.23, 1.46)	1,392 (63.2)/1,254 (56.1)	1.35 (1.19, 1.52)	2,560 (63.4)/2,395 (56.1)	1.35 (1.24, 1.48)
91 months	2,445 (61.3)/2,281 (54.0)	1.34 (1.23, 1.47)	1,370 (62.2)/1,234 (55.2)	1.34 (1.19, 1.51)	2,397 (61.2)/2,231 (54.2)	1.33 (1.22, 1.46)
103 months	2,541 (62.8)/2,294 (54.5)	1.41 (1.29, 1.54)	1,401 (63.7)/1,228 (54.9)	1.44 (1.27, 1.62)	2,474 (62.8)/2,232 (54.6)	1.41 (1.29, 1.54)
128 months	2,450 (63.4)/2.072 (52.8)	1.55 (1.42, 1.70)	1,398 (63.5)/1,180 (52.8)	1.56 (1.38, 1.76)	2,410 (63.4)/2,019 (53.0)	1.53 (1.40, 1.68)
157 months	2,303 (65.2)/1,517 (43.2)	2.46 (2.24, 2.71)	1,401 (63.7)/948 (42.4)	2.38 (2.11, 2.68)	2,247 (65.2)/1,476 (43.3)	2.45 (2.22, 2.70)
166 months	2,462 (70.1)/1,640 (46.3)	2.71 (2.46, 2.99)	1,541 (70.0)/1,022 (45.7)	2.77 (2.45, 3.14)	2,404 (70.1)/1,579 (46.5)	2.69 (2.44, 2.98)
**F:M OR (95% CI) per year of age**		**1.08 (1.07, 1.09)**		**1.09 (1.07, 1.10)**		**1.08 (1.07, 1.09)**
P(interaction) with sex		***<0.001***		***<0.001***		***<0.001***
**Rash**						
57 months	1,258 (27.8)/1,320 (27.1)	1.03 (0.94, 1.13)	631 (29.0)/628 (28.2)	1.04 (0.91, 1.18)	1,237 (27.8)/1,274 (26.9)	1.05 (0.96, 1.15)
69 months	917 (22.0)/911 (20.6)	1.09 (0.98, 1.21)	515 (23.7)/486 (21.8)	1.11 (0.96, 1.28)	891 (21.9)/877 (20.4)	1.09 (0.99, 1.22)
81 months	854 (20.7)/742 (17.0)	1.28 (1.14, 1.42)	464 (21.3)/398 (17.9)	1.24 (1.07, 1.44)	833 (20.6)/718 (16.8)	1.28 (1.15, 1.43)
91 months	749 (18.8)/638 (15.1)	1.30 (1.16, 1.46)	435 (20.0)/359 (16.1)	1.30 (1.11, 1.51)	730 (18.7)/622 (15.1)	1.29 (1.15, 1.45)
103 months	822 (20.3)/631 (15.0)	1.45 (1.29, 1.62)	445 (20.4)/339 (15.2)	1.43 (1.22, 1.67)	795 (20.2)/615 (15.1)	1.43 (1.27, 1.60)
128 months	753 (19.5)/574 (14.6)	1.42 (1.26, 1.60)	429 (19.7)/327 (14.7)	1.42 (1.22, 1.67)	735 (19.4)/561 (14.7)	1.39 (1.23, 1.57)
157 months	582 (16.6)/439 (12.6)	1.38 (1.21, 1.58)	380 (17.5)/298 (13.4)	1.37 (1.16, 1.61)	562 (16.4)/424 (12.5)	1.37 (1.20, 1.57)
166 months	631 (18.0)/445 (12.6)	1.53 (1.34, 1.74)	386 (17.7)/303 (13.6)	1.37 (1.16, 1.61)	613 (17.9)/425 (12.5)	1.52 (1.33, 1.74)
**F:M OR (95% CI) per year of age**		**1.04 (1.03, 1.06)**		**1.03 (1.01, 1.05)**		**1.04 (1.03, 1.06)**
P(interaction) with sex		***<0.001***		***0.001***		***<0.001***
**Wheezing**						
57 months	613 (13.5)/917 (18.8)	0.68 (0.60, 0.76)	268 (12.2)/360 (16.1)	0.72 (0.61, 0.86)	598 (13.5)/884 (18.7)	0.68 (0.61, 0.76)
69 months	509 (12.2)/700 (15.8)	0.74 (0.66, 0.84)	246 (11.2)/320 (14.3)	0.75 (0.63, 0.90)	501 (12.3)/677 (15.7)	0.75 (0.66, 0.85)
81 months	440 (10.7)/600 (13.7)	0.75 (0.66, 0.86)	217 (9.8)/288 (12.9)	0.74 (0.61, 0.89)	429 (10.6)/588 (13.8)	0.74 (0.65, 0.85)
91 months	353 (8.8)/531 (12.6)	0.68 (0.59, 0.78)	192 (8.7)/261 (11.6)	0.72 (0.59, 0.88)	343 (8.8)/516 (12.5)	0.67 (0.58, 0.78)
103 months	356 (8.8)/557 (13.2)	0.63 (0.55, 0.73)	185 (8.4)/282 (12.6)	0.64 (0.52, 0.77)	350 (8.9)/551 (13.5)	0.63 (0.54, 0.72)
128 months	333 (8.6)/490 (12.5)	0.66 (0.57, 0.77)	185 (8.4)/260 (11.6)	0.70 (0.57, 0.85)	328 (8.6)/478 (12.5)	0.66 (0.57, 0.76)
157 months	311 (8.8)/432 (12.4)	0.69 (0.59, 0.80)	192 (8.7)/262 (11.7)	0.72 (0.59, 0.88)	303 (8.8)/419 (12.4)	0.69 (0.59, 0.80)
166 months	288 (8.2)/372 (10.5)	0.76 (0.65, 0.90)	180 (8.2)/228 (10.2)	0.78 (0.64, 0.96)	281 (8.2)/365 (10.7)	0.74 (0.63, 0.87)
**F:M OR (95% CI) per year of age**		**1.00 (0.98, 1.02)**		**1.00 (0.98, 1.02)**		**1.00 (0.98, 1.02)**
P(interaction) with sex		***0.93***		***0.92***		***0.86***

Supplementary Table 4 continued

	Cross-sectional samples		Longitudinal sample		Mother-only cross-sectional samples	
	N (%) outcome females / males	OR (95% CI)	N (%) outcome females / males	OR (95% CI)	N (%) outcome females / males	OR (95% CI)
**Breathlessness**						
57 months	304 (6.7)/498 (10.2)	0.63 (0.54, 0.73)	118 (5.4)/191 (8.5)	0.61 (0.48, 0.77)	298 (6.7)/481 (10.2)	0.64 (0.55, 0.74)
69 months	255 (6.1)/373 (8.4)	0.71 (0.60, 0.84)	112 (5.1)/164 (7.3)	0.68 (0.53, 0.87)	252 (6.2)/360 (8.4)	0.72 (0.61, 0.86)
81 months	219 (5.3)/331 (7.6)	0.68 (0.57, 0.82)	109 (5.0)/144 (6.4)	0.76 (0.59, 0.98)	217 (5.4)/326 (7.6)	0.69 (0.57, 0.82)
91 months	188 (4.7)/300 (7.1)	0.65 (0.54, 0.78)	99 (4.5)/140 (6.3)	0.71 (0.54, 0.92)	183 (4.7)/288 (7.0)	0.65 (0.54, 0.79)
103 months	249 (0.62)/341 (8.1)	0.74 (0.63, 0.88)	118 (5.4)/170 (7.6)	0.69 (0.54, 0.88)	241 (6.1)/337 (8.3)	0.73 (0.61, 0.86)
128 months	237 (6.1)/353 (9.0)	0.66 (0.56, 0.79)	131 (6.0)/174 (7.8)	0.75 (0.60, 0.95)	236 (6.2)/344 (9.0)	0.67 (0.56, 0.79)
157 months	273 (7.8)/287 (8.2)	0.94 (0.79, 1.12)	164 (7.5)/170 (7.6)	0.98 (0.79, 1.23)	267 (7.8)/274 (8.1)	0.96 (0.81, 1.14)
166 months	252 (7.2)/244 (6.9)	1.05 (0.87, 1.26)	147 (6.7)/146 (6.5)	1.03 (0.81, 1.30)	245 (7.2)/240 (7.1)	1.01 (0.84, 1.22)
**F:M OR (95% CI) per year of age**		**1.05 (1.03, 1.07)**		**1.05 (1.02, 1.08)**		**1.05 (1.02, 1.07)**
P(interaction) with sex		***<0.001***		***<0.001***		***<0.001***
**Headache**						
57 months	1,515 (33.3)/1,505 (30.8)	1.12 (1.03, 1.22)	712 (32.1)/682 (30.2)	1.09 (0.96, 1.24)	1,491 (33.4)/1,459 (30.7)	1.13 (1.04, 1.23)
69 months	1,285 (30.9)/1,291 (29.1)	1.09 (0.99, 1.19)	682 (30.8)/697 (30.9)	0.99 (0.87, 1.13)	1,255 (30.8)/1,252 (29.1)	1.09 (0.99, 1.19)
81 months	1,694 (41.0)/1,723 (39.4)	1.07 (0.98, 1.17)	936 (42.2)/925 (41.0)	1.05 (0.93, 1.18)	1.667 (41.3)/1,680 (39.4)	1.08 (0.99, 1.18)
91 months	1,919 (48.1)/1,873 (44.3)	1.17 (1.07, 1.27)	1,100 (49.6)/1,041 (46.1)	1.15 (1.02, 1.29)	1,886 (48.2)/1,836 (44.6)	1.16 (1.06, 1.27)
103 months	2,168 (53.6)/2,142 (50.9)	1.11 (1.02, 1.21)	1,220 (55.0)/1,176 (52.1)	1.12 (1.00, 1.26)	2,115 (53.7)/1,086 (51.0)	1.11 (1.02, 1.22)
128 months	2,669 (69.0)/2,480 (63.1)	1.30 (1.18, 1.43)	1,554 (70.1)/1,439 (63.8)	1.33 (1.17, 1.51)	2,620 (68.8)/2,417 (63.4)	1.27 (1.16, 1.40)
157 months	2,704 (76.2)/2,412 (68.4)	1.48 (1.33, 1.64)	1,685 (76.0)/1,561 (69.2)	1.41 (1.23, 1.61)	2,645 (76.3)/2,348 (68.6)	1.47 (1.32, 1.64)
166 months	2,807 (79.8)/2,496 (70.4)	1.66 (1.49, 1.86)	1,784 (80.4)/1,602 (71.0)	1.68 (1.46, 1.93)	2,744 (79.9)/2,409 (70.9)	1.64 (1.46, 1.83)
**F:M OR (95% CI) per year of age**		**1.05 (1.03, 1.06)**		**1.05 (1.04, 1.07)**		**1.04 (1.03, 1.06)**
P(interaction) with sex		***<0.001***		***<0.001***		***<0.001***
**Constipation**						
57 months	698 (15.4)/659 (13.5)	1.16 (1.04, 1.31)	324 (14.8)/281 (12.6)	1.21 (1.02, 1.44)	684 (15.3)/643 (13.5)	1.16 (1.03, 1.30)
69 months	466 (11.2)/421 (9.5)	1.20 (1.05, 1.38)	242 (11.1)/194 (8.7)	1.31 (1.07, 1.60)	456 (11.3)/405 (9.4)	1.22 (1.06, 1.40)
81 months	490 (11.9)/389 (8.9)	1.38 (1.20, 1.59)	274 (12.5)/193 (8.6)	1.52 (1.25, 1.84)	483 (12.0)/379 (8.9)	1.39 (1.21, 1.61)
91 months	439 (11.0)/388 (9.2)	1.22 (1.06, 1.41)	237 (10.9)/198 (8.9)	1.25 (1.03, 1.53)	430 (11.0)/383 (9.3)	1.21 (1.04, 1.40)
103 months	438 (10.9)/385 (9.2)	1.21 (1.05, 1.40)	227 (10.4)/185 (8.3)	1.28 (1.05, 1.57)	424 (10.8)/373 (9.1)	1.20 (1.04, 1.39)
128 months	410 (10.7)/347 (8.8)	1.23 (1.06, 1.43)	220 (10.1)/191 (8.6)	1.20 (0.98, 1.47)	400 (10.6)/333 (8.7)	1.23 (1.06, 1.44)
157 months	301 (8.6)/270 (7.8)	1.12 (0.94, 1.33)	167 (7.6)/167 (7.5)	1.02 (0.82, 1.28)	287 (8.4)/263 (7.8)	1,08 (0.91, 1.29)
166 months	277 (7.9)/259 (7.3)	1.09 (0.91, 1.30)	163 (7.5)/154 (6.9)	1.09 (0.87, 1.37)	270 (7.9)/252 (7.4)	1.07 (0.89, 1.28)
**F:M OR (95% CI) per year of age**		**0.99 (0.97, 1.01)**		**0.98 (0.95, 1.00)**		**0.99 (0.97, 1.01)**
P(interaction) with sex		***0.39***		***0.07***		***0.26***

Supplementary Table 4 continued

	Cross-sectional samples		Longitudinal sample		Mother-only cross-sectional samples	
	N (%) outcome females / males	OR (95% CI)	N (%) outcome females / males	OR (95% CI)	N (%) outcome females / males	OR (95% CI)
**Head lice / scabies**						
57 months	843 (18.5)/565 (11.6)	1.74 (1.55, 1.95)	376 (17.0)/239 (10.6)	1.73 (1.45, 2.05)	830 (18.6)/550 (11.6)	1.75 (1.55, 1.96)
69 months	1,356 (32.7)/824 (18.7)	2.12 (1.92, 2.34)	692 (31.3)/409 (18.2)	2.05 (1.78, 2.36)	1,328 (32.7)/797 (18.6)	2.13 (1.93, 2.36)
81 months	2,408 (58.3)/1,571 (35.9)	2.50 (2.29, 2.73)	1,243 (56.3)/780 (34.7)	2.42 (2.15, 2.74)	2,366 (58.6)/1,525 (35.7)	2.54 (2.33, 2.78)
91 months	2,408 (60.3)/1,546 (36.6)	2.64 (2.41, 2.88)	1,306 (59.2)/786 (35.0)	2.69 (2.39, 3.04)	2,346 (60.3)/1,507 (36.5)	2.64 (2.42, 2.89)
103 months	2,427 (60.0)/1.410 (33.5)	2.98 (2.73, 3.26)	1,294 (58.6)/738 (32.8)	2.90 (2.56, 3.27)	2,359 (59.9)/1,367 (33.4)	2.98 (2.72, 3.26)
128 months	1,822 (47.1)/770 (19.6)	3.65 (3.30, 4.03)	996 (45.1)/429 (19.1)	3.48 (3.05, 3.99)	1,795 (47.1)/745 (19.6)	3.67 (3.31, 4.06)
157 months	791 (22.5)/313 (8.9)	2.95 (2.57, 3.40)	500 (22.6)/182 (8.1)	3.32 (2.77, 3.98)	774 (22.5)/307 (9.0)	2.93 (2.54, 3.37)
166 months	593 (16.9)/270 (7.6)	2.47 (2.12, 2.87)	371 (16.8)/160 (7.1)	2.64 (2.17, 3.21)	580 (16.9)/261 (7.7)	2.45 (2.10, 2.86)
**F:M OR (95% CI) per year of age**		**1.03 (1.02, 1.04)**		**1.04 (1.02, 1.05)**		**1.03 (1.02, 1.04)**
P(interaction) with sex		***<0.001***		***<0.001***		***<0.001***
**Eczema**						
81 months	724 (17.5)/639 (14.6)	1.24 (1.11, 1.40)	419 (18.2)/377 (16.1)	1.16 (0.99, 1.35)	711 (17.6)/625 (14.7)	1.24 (1.11, 1.40)
91 months	708 (17.8)/632 (15.0)	1.23 (1.09, 1.38)	437 (18.9)/374 (15.9)	1.23 (1.06, 1.43)	698 (17.9)/624 (15.1)	1.22 (1.08, 1.37)
103 months	699 (17.3)/580 (13.8)	1.31 (1.16, 1.47)	434 (18.8)/340 (14.5)	1.37 (1.17, 1.60)	680 (17.3)/571 (14.0)	1.29 (1.14, 1.45)
128 months	649 (16.8)/543 (13.8)	1.26 (1.11, 1.42)	401 (17.4)/346 (14.7)	1.22 (1.04, 1.42)	640 (16.8)/532 (14.0)	1.24 (1.10, 1.41)
157 months	485 (3.8)/422 (12.1)	1.16 (1.01, 1.34)	330 (14.3)/296 (12.6)	1.16 (0.98, 1.37)	472 (13.8)/416 (12.3)	1.14 (0.99, 1.31)
166 months	482 (13.8)/432 (11.9)	1.18 (1.02, 1.35)	328 (14.2)/303 (12.9)	1.12 (0.94, 1.32)	474 (13.8)/413 (12.1)	1.16 (1.01, 1.34)
**F:M OR (95% CI) per year of age**		**0.99 (0.97, 1.01)**		**0.99 (0.97, 1.01)**		**0.99 (0.97, 1.01)**
P(interaction) with sex		***0.31***		***0.30***		***0.23***
**Asthma**						
81 months	454 (11.0)/609 (13.9)	0.76 (0.67, 0.87)	221 (9.5)/297 (12.7)	0.73 (0.60, 0.87)	441 (10.9)/594 (13.9)	0.75 (0.66, 0.86)
91 months	387 (9.7)/568 (13.4)	0.69 (0.60, 0.79)	208 (9.0)/283 (12.1)	0.72 (0.59, 0.87)	377 (9.6)/554 (13.4)	0.69 (0.60, 0.79)
103 months	402 (10.0)/572 (13.7)	0.70 (0.61, 0.80)	217 (9.3)/299 (12.8)	0.71 (0.59, 0.85)	395 (10.1)/563 (13.8)	0.70 (0.61, 0.80)
128 months	389 (10.1)/546 (13.9)	0.69 (0.60, 0.79)	230 (9.9)/294 (12.5)	0.77 (0.64, 0.92)	383 (10.1)/532 (14.0)	0.69 (0.60, 0.79)
157 months	360 (10.2)/473 (13.5)	0.73 (0.63, 0.84)	228 (9.8)/298 (12.7)	0.75 (0.62, 0.90)	352 (10.2)/460 (13.6)	0.73 (0.63, 0.84)
166 months	366 (10.4)/452 (12.8)	0.80 (0.69, 0.92)	234 (10.1)/292 (12.5)	0.79 (0.66, 0.95)	359 (10.5)/440 (13.0)	0.79 (0.68, 0.91)
**F:M OR (95% CI) per year of age**		**1.01 (0.99, 1.03)**		**1.01 (0.99, 1.04)**		**1.01 (0.99, 1.03)**
P(interaction) with sex		***0.54***		***0.37***		***0.54***

Supplementary Table 4 continued

	Cross-sectional samples		Longitudinal sample		Mother-only cross-sectional samples	
	**N (%) outcome females / males**	**OR (95% CI)**	**N (%) outcome females / males**	**OR (95% CI)**	**N (%) outcome females / males**	**OR (95% CI)**
**Hay fever**						
81 months	208 (5.0)/328 (7.5)	0.65 (0.55, 0.78)	110 (4.8)/173 (7.4)	0.63 (0.49, 0.80)	202 (5.0)/324 (7.6)	0.64 (0.53, 0.77)
91 months	281 (7.1)/442 (10.5)	0.65 (0.55, 0.76)	171 (7.5)/255 (11.0)	0.65 (0.53, 0.80)	273 (7.0)/435 (10.6)	0.63 (0.54, 0.74)
103 months	442 (11.0)/604 (14.5)	0.73 (0.64, 0.84)	250 (10.9)/348 (15.0)	0.69 (0.58, 0.83)	433 (11.1)/586 (14.4)	0.74 (0.65, 0.84)
128 months	523 (13.6)/650 (16.6)	0.79 (0.70, 0.89)	326 (14.2)/410 (17.6)	0.77 (0.66, 0.91)	512 (13.5)/636 (16.8)	0.78 (0.68, 0.88)
157 months	601 (17.1)/781 (22.4)	0.72 (0.64, 0.81)	394 (17.2)/523 (22.5)	0.71 (0.62, 0.83)	583 (17.0)/758 (22.4)	0.71 (0.63, 0.80)
166 months	655 (18.7)/830 (23.4)	0.75 (0.67, 0.84)	428 (18.7)/560 (24.1)	0.72 (0.63, 0.83)	644 (18.8)/802 (23.6)	0.75 (0.67, 0.84)
**F:M OR (95% CI) per year of age**		**1.01 (0.99, 1.03)**		**1.01 (0.99, 1.04)**		**1.01 (0.99, 1.03)**
P(interaction) with sex		***0.21***		***0.31***		***0.18***
**Pains in arms / legs**						
57 months	650 (14.4)/732 (15.1)	0.95 (0.85, 1.07)	305 (13.8)/330 (14.6)	0.93 (0.79, 1.11)	642 (14.5)/713 (15.1)	0.96 (0.85, 1.08)
69 months	732 (17.6)/786 (17.8)	0.99 (0.88, 1.10)	381 (17.3)/409 (18.1)	0.94 (0.81, 1.10)	715 (17.6)/774 (18.0)	0.97 (0.87, 1.09)
81 months	736 (18.2)/832 (19.5)	0.92 (0.82, 1.03)	386 (17.5)/443 (19.6)	0.87 (0.74, 1.01)	723 (18.3)/825 (19.9)	0.90 (0.81, 1.01)
103 months	857 (21.3)/908 (21.8)	0.97 (0.88, 1.08)	451 (20.4)/478 (21.2)	0.95 (0.83, 1.10)	842 (21.5)/893 (22.0)	0.97 (0.87, 1.08)
140 months	1,003 (27.4)/1,151 (31.7)	0.82 (0.74, 0.90)	592 (26.8)/674 (29.9)	0.86 (0.75, 0.98)	983 (27.3)/1,134 (31.8)	0.81 (0.73, 0.89)
157 months	1,026 (30.2)/1,173 (34.7)	0.81 (0.74, 0.90)	689 (31.2)/782 (34.7)	0.85 (0.75, 0.97)	1,006 (30.3)/1,146 (34.9)	0.81 (0.73, 0.90)
**F:M OR (95% CI) per year of age**		**0.98 (0.96, 0.99)**		**0.99 (0.97, 1.01)**		**0.98 (0.96, 0.99)**
P(interaction) with sex		***0.003***		***0.29***		***0.003***
**Food allergy**						
65 months	349 (8.0)/425 (9.2)	0.86 (0.74, 1.00)	183 (7.4)/237 (9.4)	0.77 (0.63, 0.95)	344 (8.1)/413 (9.1)	0.87 (0.75, 1.01)
81 months	373 (9.8)/422 (10.4)	0.93 (0.80, 1.08)	223 (9.0)/267 (10.5)	0.84 (0.70, 1.02)	368 (9.9)/4121 (10.4)	0.94 (0.81, 1.09)
103 months	224 (5.5)/273 (6.5)	0.85 (0.71, 1.02)	123 (5.)/145 (5.7)	0.86 (0.67, 1.10)	220 (5.6)/268 (6.5)	0.84 (0.70, 1.02)
157 months	341 (9.6)/347 (9.8)	0.97 (0.83, 1.14)	233 (9.4)/260 (10.3)	0.91 (0.76, 1.10)	335 (9.7)/339 (9.9)	0.97 (0.83, 1.14)
**F:M OR (95% CI) per year of age**		**1.01 (0.99, 1.04)**		**1.02 (0.99, 1.05)**		**1.01 (0.99, 1.04)**
P(interaction) with sex		***0.23***		***0.16***		***0.30***
**Other allergy**						
65 months	514 (12.3)/576 (13.0)	0.94 (0.82, 1.06)	276 (11.8)/310 (12.9)	0.90 (0.76, 1.07)	506 (12.3)/568 (13.2)	0.93 (0.82, 1.06)
81 months	556 (14.1)/635 (15.3)	0.91 (0.81, 1.03)	312 (13.3)/368 (15.3)	0.85 (0.72, 1.00)	547 (14.2)/625 (15.4)	0.91 (0.80, 1.03)
103 months	856 (21.5)/973 (23.5)	0.89 (0.80, 0.99)	509 (21.8)/570 (23.7)	0.89 (0.78, 1.02)	838 (21.6)/956 (23.8)	0.88 (0.79, 0.98)
157 months	925 (27.1)/954 (27.9)	0.96 (0.86, 1.07)	612 (26.2)/666 (27.7)	0.92 (0.81, 1.05)	904 (27.1)/934 (28.2)	0.95 (0.85, 1.05)
**F:M OR (95% CI) per year of age**		**1.00 (0.99, 1.02)**		**1.01 (0.99, 1.03)**		**1.00 (0.98, 1.02)**
P(interaction) with sex		***0.63***		***0.54***		***0.71***

Supplementary Table 5: OR (95% CI) for infections in females compared with males

	Cross-sectional samples		Longitudinal sample		Mother-only cross-sectional samples	
	**N (%) outcome females / males**	**OR (95% CI)**	**N (%) outcome females / males**	**OR (95% CI)**	**N (%) outcome females / males**	**OR (95% CI)**
**Chicken pox**						
57 months	1,723 (38.1)/1,949 (40.2)	0.91 (0.84, 0.99)	836 (37.4)/904 (39.3)	0.92 (0.82, 1.04)	1,690 (38.1)/1,889(40.1)	0.92 (0.84, 1.00)
69 months	662 (15.9)/777 (17.6)	0.89 (0.79, 0.99)	351 (15.7)/3987 (17.3)	0.89 (0.76, 1.04)	652 (16.0)/762 (17.8)	0.89 (0.79, 0.99)
81 months	319 (7.8)/322 (7.4)	1.05 (0.90, 1.24)	173 (7.7)/158 (6.9)	1.13 (0.91, 1.42)	312 (7.8)/309 (7.3)	1.07 (0.91, 1.26)
91 months	152 (3.8)/148 (3.5)	1.09 (0.87, 1.37)	78 (3.5)/63 (2.7)	1.28 (0.91, 1.79)	150 (3.8)/146 (3.5)	1.08 (0.86, 1.37)
103 months	66 (1.6)/70 (1.7)	0.98 (0.70, 1.38)	32 (1.4)/30 (1.3)	1.10 (0.66, 1.81)	66 (1.7)/68 (1.7)	1.01 (0.72, 1.42)
128 months	34 (0.9)/20 (0.5)	1.74 (1.00, 3.02)	16 (0.7)/10 (0.4)	1.65 (0.75, 3.64)	33 (0.9)/20 (0.5)	1.66 (0.95, 2.90)
166 months	12 (0.4)/15 (0.4)	0.81 (0.38, 1.74)	7 (0.3)/8 (0.4)	0.90 (0.33, 2.48)	11 (0.3)/15 (0.5)	0.73 (0.34, 1.60)
**F:M OR (95% CI) per year of age**		**1.07 (1.00, 1.14)**		**1.10 (0.99, 1.21)**		**1.06 (0.99, 1.14)**
P(interaction) with sex		***0.07***		***0.07***		***0.09***
**Cold sores**						
57 months	328 (7.7)/319 (6.6)	1.18 (1.01, 1.38)	157 (7.0)/130 (5.7)	1.25 (0.99, 1.59)	338 (7.7)/307 (6.6)	1.18 (1.01, 1.39)
69 months	284 (6.9)/259 (5.9)	1.18 (0.99, 1.40)	152 (6.8)/125 (5.5)	1.26 (0.99, 1.61)	275 (6.8)/251 (5.9)	1.17 (0.98, 1.39)
81 months	269 (6.5)/265 (6.1)	1.08 (0.91, 1.29)	140 (6.3)/124 (5.4)	1.17 (0.91, 1.49)	264 (6.6)/260 (6.1)	1.08 (0.90, 1.29)
91 months	299 (7.5)/254 (6.0)	1.27 (1.06, 1.50)	142 (6.4)/118 (5.2)	1.25 (0.97, 1.60)	287 (7.3)/249 (6.0)	1.23 (1.03, 1.47)
103 months	275 (6.8)/233 (5.5)	1.24 (1.04, 1.49)	139 (6.2)113 (5.0)	1.28 (0.99, 1.65)	265 (6.7)/226 (5.5)	1.23 (1.03, 1.48)
128 months	298 (7.9)/221 (5.7)	1.40 (1.17, 1.68)	152 (6.8)/119 (5.2)	1.33 (1.04, 1.70)	292 (7.8)/212 (5.7)	1.41 (1.18, 1.70)
166 months	298 (8.5)/188 (5.4)	1.64 (1.36, 1.98)	166 (7.4)/108 (4.7)	1.62 (1.26, 2.08)	282 (8.4)/178 (5.3)	1.64 (1.35, 1.99)
**F:M OR (95% CI) per year of age**		**1.04 (1.02, 1.07)**		**1.03 (1.00, 1.06)**		**1.04 (1.02, 1.07)**
P(interaction) with sex		***<0.001***		***0.05***		***0.001***
**Eye infection**						
57 months	689 (15.3)/736 (15.3)	1.00 (0.90, 1.12)	369 (15.9)/380 (16.1)	0.99 (0.84, 1.15)	684 (15.5)/714 (15.3)	1.02 (0.91, 1.14)
69 months	338 (8.2)/349 (7.9)	1.03 (0.88, 1.21)	188 (8.1)/195(8.3)	0.98 (0.79, 1.21)	332 (8.2)/329 (7.7)	1.07 (0.91, 1.25)
81 months	195 (4.7)/188 (4.3)	1.10 (0.90, 1.35)	111 (4.8)/108 (4.6)	1.05 (0.80, 1.37)	193 (4.8)/180 (4.2)	1.14 (0.93, 1.40)
91 months	141 (3.5)/134 (3.2)	1.12 (0.88, 1.42)	76 (3.3)/73 (3.1)	1.06 (0.77, 1.47)	138 (3.5)/133 (3.2)	1.09 (0.86, 1.39)
103 months	151 (3.7)/110 (2.6)	1.44 (1.13, 1.85)	93 (4.0)/61 (2.6)	1.57 (1.13, 2.18)	145 (3.7)/106 (2.6)	1.44 (1.11, 1.85)
128 months	149 (3.9)/131 (3.4)	1.17 (0.92, 1.48)	79 (3.4)/72 (3.1)	1.12 (0.81, 1.55)	146 (3.9)/128 (3.4)	1.15 (0.90, 1.47)
166 months	185 (5.3)/1.03 (2.9)	1.87 (1.46, 2.39)	108 (4.7)/60 (2.6)	1.87 (1.36, 2.58)	178 (5.2)/101 (3.0)	1.79 (1.40, 2.30)
**F:M OR (95% CI) per year of age**		**1.11 (1.06, 1.15)**		**1.11 (1.05, 1.17)**		**1.10 (1.05, 1.14)**
P(interaction) with sex		***<0.001***		***<0.001***		***<0.001***

Supplementary Table 5 continued

	Cross-sectional samples		Longitudinal sample		Mother-only cross-sectional samples	
	N (%) outcome females / males	OR (95% CI)	N (%) outcome females / males	OR (95% CI)	N (%) outcome females / males	OR (95% CI)
**Ear infection**						
57 months	1,507 (33.4)/1,605 (33.2)	1.01 (0.93, 1.10)	762 (33.0)/776 (32.8)	1.01 (0.89, 1.14)	1,483 (33.5)/1,571 (33.4)	1.01 (0.92, 1.10)
69 months	1,099 (26.5)/1,103 (25.0)	1.08 (0.98, 1.19)	603 (26.1)/592 (25.1)	1.06 (0.93, 1.21)	1,084 (26.8)/1,072 (25.1)	1.09 (0.99, 1.20)
81 months	808 (19.6)/773 (17.8)	1.13 (1.02, 1.26)	447 (19.4)/407 (17.2)	1.15 (1.00, 1.34)	794 (19.7)/755 (17.8)	1.14 (1.02, 1.27)
91 months	613 (15.4)/535 (12.7)	1.25 (1.10, 1.42)	337 (14.60/285 (12.1)	1.25 (1.05, 1.48)	603 (15.4)/524 (12.7)	1.25 (1.10, 1.42)
103 months	459 (11.3)/385 (9.2)	1.27 (1.10, 1.46)	245 (10.6)/210 (8.9)	1.22 (1.00, 1.48)	446 (11.3)/377 (9.2)	1.26 (1.09, 1.45)
128 months	363 (9.5)/255 (6.5)	1.50 (1.27, 1.77)	206 (8.9)/142 (6.0)	1.53 (1.23, 1.91)	355 (9.4)/245 (6.5)	1.50 (1.27, 1.78)
166 months	236 (6.8)/212 (6.0)	1.14 (0.94, 1.38)	151 (6.6)/138 (5.8)	1.13 (0.89, 1.43)	233 (6.8)/202 (6.0)	1.16 (0.95, 1.41)
**F:M OR (95% CI) per year of age**		**1.05 (1.02, 1.07)**		**1.04 (1.01, 1.08)**		**1.05 (1.02, 1.07)**
P(interaction) with sex		***<0.001***		***0.01***		***<0.001***
**Chest infection**						
57 months	977 (21.7)/1,190 (24.7)	0.85 (0.77, 0.93)	446 (19.5)/518 (22.3)	0.85 (0.73, 0.98)	960 (21.8)/1,154 (24.6)	0.85 (0.77, 0.94)
69 months	653 (15.9)/771 (17.6)	0.88 (0.79, 0.99)	324 (14.2)/371 (16.0)	0.87 (0.74, 1.02)	641 (16.0)/744 (17.5)	0.90 (0.80, 1.01)
81 months	431 (10.5)/ 475 (10.9)	0.96 (0.83, 1.10)	217 (9.5)/253 (10.9)	0.86 (0.71, 1.04)	421 (10.5)/467 (11.0)	0.95 (0.82, 1.09)
91 months	220 (5.5)/293 (7.0)	0.78 (0.65, 0.94)	110 (4.8)/143 (6.2)	0.77 (0.60, 1.00)	214 (5.5)/281 (6.8)	0.79 (0.66, 0.95)
103 months	193 (4.8)/244 (5.8)	0.81 (0.67, 0.98)	103 (4.5)/131 (5.6)	0.79 (0.61, 1.03)	191 (4.9)/241 (5.9)	0.81 (0.67, 0.99)
128 months	192 (5.0)/228 (5.8)	0.85 (0.70, 1.04)	98 (4.3)/115 (5.0)	0.86 (0.65, 1,14)	188 (5.0)/217 (5.7)	0.86 (0.71, 1.06)
166 months	174 (5.0)/202 (5.7)	0.87 (0.70, 1.07)	108 (4.7)/116 (5.0)	0.95 (0.72, 1.24)	169 (5.0)/190 (5.6)	0.88 (0.71, 1.09)
**F:M OR (95% CI) per year of age**		**1.00 (0.97, 1.03)**		**1.01 (0.97, 1.045**		**1.00 (0.97, 1.03)**
P(interaction) with sex		***0.91***		***0.75***		***0.99***
**Tonsilitis / laryngitis**						
91 months	320 (8.0)/288 (6.8)	1.19 (1.01, 1.41)	187 (7.3)/176 (6.7)	1.10 (0.89, 1.36)	315 (8.1)/282 (6.9)	1.19 (1.01, 1.41)
103 months	299 (7.4)/214 (5.1)	1.49 (1.24, 1.79)	185 (7.2)/125 (4.7)	1.56 (1.24, 1.97)	294 (7.5)/213 (5.2)	1.47 (1.22, 1.76)
128 months	348 (9.1)/221 (5.7)	1.67 (1.40, 1.98)	224 (8.7)/138 (5.2)	1.73 (1.39, 2.16)	341 (9.0)/215 (5.7)	1.65 (1.38, 1.97)
166 months	362 (10.4)/267 (7.6)	1.41 (1.20, 1.67)	251 (9.8)/182 (6.9)	1.46 (1.20, 1.78)	353 (10.4)/253 (7.5)	1.43 (1.21, 1.69)
**F:M OR (95% CI) per year of age**		**1.02 (0.99, 1.06)**		**1.03 (0.99, 1.08)**		**1.02 (0.99, 1.06)**
P(interaction) with sex		***0.24***		***0.14***		***0.19***

Supplementary Table 5 continued

	Cross-sectional samples		Longitudinal sample		Mother-only cross-sectional samples	
	**N (%) outcome females / males**	**OR (95% CI)**	**N (%) outcome females / males**	**OR (95% CI)**	**N (%) outcome females / males**	**OR (95% CI)**
**Influenza**						
91 months	223 (5.6)/214 (5.1)	1.11 (0.91, 1.34)	139 (5.4)/130 (5.0)	1.09 (0.85, 1.39)	216 (5.5)/209 (5.1)	1.09 (0.90, 1.33)
103 months	167 (4.1)/173 (4.1)	1.00 (0.81, 1.25)	85 (3.3)/93 (3.6)	0.93 (0.69, 1.25)	162 (4.1)/166 (4.1)	1.01 (0.81, 1.26)
128 months	196 (5.1)/176 (4.5)	1.14 (0.92, 1.40)	116 (4.5)/104 (4.0)	1.14 (0.87, 1.49)	189 (5.0)/168 (4.5)	1.13 (0.92, 1.40)
166 months	235 (6.8)/230 (6.5)	1.04 (0.86, 1.26)	147 (5.8)/151 (5.9)	0.99 (0.79, 1.26)	226 (6.7)/217 (6.4)	1.04 (0.86, 1.27)
**F:M OR (95% CI) per year of age**		**1.00 (0.96, 1.04)**		**0.99 (0.94, 1.05)**		**1.00 (0.96, 1.04)**
P(interaction) with sex		***0.88***		***0.81***		***0.94***
**Cold**						
57 months	4,406 (97.0)/4,687 (96.1)	1.31 (1.04, 1.63)	2,235 (97.5)/2,265 (97.4)	1.04 (0.72, 1.49)	4,326 (97.0)/4,558 (96.2)	1.30 (1.04, 1.63)
69 months	3,805 (93.0)/4,014 (91.7)	1.20 (1.02, 1.41)	2,164(94.4)/2,173 (93.4)	1.18 (0.93, 1.50)	3,718 (93.0)/3,894 (91.7)	1.20 (1.02, 1.41)
81 months	3,639 (88.2)/3,774 (86.3)	1.18 (1.04, 1.34)	2,063 (90.0)/2,055 (88.4)	1.18 (0.98, 1.42)	3,561 (88.2)/3,689 (86.5)	1.17 (1.03, 1.33)
91 months	2,687 (93.7)/3,814 (91.9)	1.31 (1.11, 1.56)	2,157 (94.1)/2,157 (92.7)	1.24 (0.98, 1.57)	3,622 (93.8)/3,730 (92.0)	1.31 (1.10, 1.55)
103 months	3,695 (91.1)/3,773 (89.6)	1.19 (1.03, 1.38)	2,106 (91.8)/2,114 (90.9)	1.13 (0.92, 1.39)	3,596 (91.0)/3,666 (89.6)	1.19 (1.02, 1.38)
128 months	3,174 (82.2)/3,036 (77.6)	1.34 (1.20, 1.50)	1,910 (83.3)/1,839 (79.1)	1.32 (1.14, 1.53)	3,125 (82.3)/2,953 (77.8)	1.33 (1.18, 1.48)
166 months	3.022 (86.2)/2,905 (82.0)	1.36 (1.20, 1.55)	1,993 (86.9)/1,955 (84.1)	1.26 (1.07, 1.49)	2,952 (86.2)/2,795 (82.3)	1.34 (1.18, 1.53)
**F:M OR (95% CI) per year of age**		**1.02 (1.00, 1.03)**		**1.01 (0.99, 1.04)**		**1.01 (1.00, 1.03)**
P(interaction) with sex		***0.07***		***0.24***		***0.09***
**Urinary infection**						
57 months	400 (8.9)/192 (4.0)	2.35 (1.97, 2.81)	209 (9.1)/91 (3.9)	2.49 (1.93, 3.21)	390 (8.84)/187 (4.0)	2.33 (1.95, 2.79)
69 months	271 (6.5)/117 (2.7)	2.56 (2.05, 3.19)	145 (6.3)/64 (2.7)	2.41 (1.79, 3.26)	263 (6.5)/116 (2.7)	2.49 (1.99, 3.11)
81 months	224 (5.4)/93 (2.1)	2.64 (2.06, 3.37)	109 (4.8)/47 (2.0)	2.45 (1.73, 3.46)	222 (5.5)/92 (2.2)	2.64 (2.06, 3.38)
91 months	172 (4.3)/75 (1.8)	2.50 (1.90, 3.29)	90 (3.9)/38 (1.6)	2.49 (1.69, 3.65)	169 (4.3)/74 (1.8)	2.47 (1.87, 3.26)
103 months	176 (4.4)/55 (1.3)	3.44 (2.53, 4.67)	73 (3.2)/33 (1.4)	2.31 (1.52, 3.50)	173 (4.4)/55 (1.3)	3.37 (2.48, 4.58)
128 months	138 (3.6)/41 (1.1)	3.52 (2.48, 5.00)	75 (3.3)/26 (1.1)	3.02 (1.93, 4.74)	138 (3.7)/40 (1.1)	3,56 (2.49, 5.07)
166 months	118 (3.4)/24 (0.7)	5.13 (3.30, 7.98)	65 (2.8)/15 (0.6)	4.54 (2.58, 7.99)	106 (3.1)/24 (0.7)	4.51 (2.89, 7.04)
**F:M OR (95% CI) per year of age**		**1.10 (1.05, 1.16)**		**1.07 (1.00, 1.14)**		**1.09 (1.04, 1.15)**
P(interaction) with sex		***<0.001***		***0.06***		***<0.001***

Supplementary Table 5 continued

	Cross-sectional samples		Longitudinal sample		Mother-only cross-sectional samples	
	**N (%) outcome females / males**	**OR (95% CI)**	**N (%) outcome females / males**	**OR (95% CI)**	**N (%) outcome females / males**	**OR (95% CI)**
**Worm infection**						
57 months	477 (10.5)/397 (8.1)	1.33 (1.15, 1.53)	232 (10.6)/182 (8.1)	1.33 (1.09, 1.64)	472 (10.6)/386 (8.1)	1.34 (1.16, 1.54)
69 months	418 (10.1)/318 (7.2)	1.45 (1.24, 1.68)	238 (10.8)/163 (7.3)	1.55 (1.26, 1.91)	414 (10.2)/312 (7.3)	1.45 (1.24, 1.69)
81 months	473 (11.5)/340 (7.8)	1.54 (1.33, 1.78)	267 (12.2)/172 (7.7)	1.66 (1.36, 2.03)	466 (11.5)/332 (7.8)	1.55 (1.33, 1.79)
91 months	407 (10.2)/313 (7.4)	1.42 (1.22, 1.66)	238 (10.8)/160 (7.1)	1.58 (1.28, 1.95)	402 (10.3)/305 (7.4)	1.43 (1.23, 1.67)
103 months	390 (9.7)/267 (6.4)	1.58 (1.34, 1.85)	220 (10.0)/146 (6.5)	1.60 (1.28, 1.98)	381 (9.7)/263 (6.4)	1.56 (1.32, 1.84)
128 months	237 (6.1)/144 (3.7)	1.72 (1.39, 2.12)	124 (5.6)/73 (3.3)	1.77 (1.32, 2.38)	231 (6.1)/143 (3.8)	1.66 (1.34, 2.05)
157 months	81 (2.3)/66 (1.9)	1.22 (0.88, 1.70)	45 (2.1)/42 (1.9)	1.09 (0.72, 1.67)	79 (2.3)/66 (2.0)	1.19 (0.85, 1.65)
166 months	55 (1.6)/62 (1.8)	0.90 (0.62, 1.29)	31 (1.4)/38 (1.7)	0.83 (0.51, 1.34)	53 (1.6)/60 (1.8)	0.87 (0.60, 1.27)
**F:M OR (95% CI) per year of age**		**0.99 (0.97, 1.02)**		**0.98 (0.95, 1.02)**		**0.99 (0.97, 1.01)**
P(interaction) with sex		***0.61***		***0.32***		***0.39***

Supplementary Table 6: OR and RR (95% CI) for general health measures in females compared with males (cross-sectional samples)

	N (%) outcome females / males	OR (95% CI)	RR (95% CI)
**Poor general health last month**			
57	89 (2.0)/94 (2.0)	1.02 (0.76, 1.36)	1.02 (0.76, 1.36)
69	69 (1.7)/67 (1.5)	1.10 (0.79, 1.55)	1.10 (0.79, 1.54)
81	42 (1.1)/35 (0.9)	1.25 (0.80, 1.96)	1.25 (0.80, 1.96)
91	30 (0.8)/44 (1.0)	0.72 (0.45, 1.15)	0.72 (0.45, 1.15)
103	43 (1.1)/40 (1.0)	1.12 (0.72, 1.72)	1.12 (0.73, 1.72)
128	48 (1.3)/32 (0.8)	1.54 (0.98, 2.42)	1.53 (0.98, 2.40)
140	37 (1.0)/33 (0.9)	1.13 (0.70, 1.81)	1.13 (0.70, 1.80)
157	49 (1.4)/38 (1.1)	1.29 (0.84, 1.98)	1.29 (0.84, 1.97)
166	56 (1.6)/29 (0.8)	1.97 (1.26, 3.10)	1.96 (1.25, 3.06)
**F:M OR (95% CI) per year of age**		**1.06 (1.01, 1.12)**	**1.06 (1.01, 1.12)**
P(interaction) with sex		***0.03***	***0.03***
**Poor general health in last year**			
57	120 (2.7)/150 (3.2)	0.85 (0.66, 1.08)	0.85 (0.67, 1.08)
69	85 (2.1)/103 (2.4)	0.87 (0.65, 1.16)	0.87 (0.66, 1.16)
81	65 (1.7)/78 (2.0)	0.87 (0.62, 1.21)	0.87 (0.62, 1.21)
91	51 (1.3)/72 (1.7)	0.75 (0.52, 1.07)	0.75 (0.52, 1.07)
103	60 (1.5)/70 (1.7)	0.88 (0.62, 1.25)	0.88 (0.63, 1.25)
128	78 (2.1)/51 (1.4)	1.58 (1.11, 2.26)	1.57 (1.10, 2.23)
140	51 (1.4)/57 (1.6)	0.90 (0.61, 1.31)	0.90 (0.62, 1.31)
157	65 (1.9)/43 (1.3)	1.51 (1.02, 2.22)	1.50 (1.02, 2.20)
166	83 (2.5)/57 (1.7)	1.50 (1.07, 2.11)	1.49 (1.06, 2.08)
**F:M OR (95% CI) per year of age**		**1.08 (1.03, 1.13)**	**1.08 (1.03, 1.12)**
P(interaction) with sex		***0.001***	***0.001***
**Any days off school in last year**			
91	3,067 (77.6)/3,142 (75.0)	1.15 (1.04, 1.28)	1.03 (0.98, 1.09)
103	3,264 (82.5)/3,238 (79.3)	1.23 (1.10, 1.38)	1.04 (0.99, 1.09)
128	3,078 (80.5)/3,008 (77.0)	1.23 (1.10, 1.37)	1.04 (0.99, 1.10)
140	3,015 (83.5)/2,984 (82.7)	1.06 (0.94, 1.20)	1.01 (0.96, 1.06)
166	2,841 (81.8)/2,779 (79.2)	1.18 (1.05, 1.33)	1.03 (0.98, 1.09)
**F:M OR (95% CI) per year of age**		**1.00 (0.97, 1.02)**	**1.00 (0.99, 1.00)**
P(interaction) with sex		***0.68***	***0.41***

Supplementary Table 7: OR and RR (95% CI) for conditions in females compared with males (cross-sectional samples)

	N (%) outcome females / males	OR (95% CI)	RR (95% CI)
**Diarrhoea**			
57	2,634 (58.1)/2,967 (61.0)	0.89 (0.82, 0.96)	0.95 (0.90, 1.00)
69	1,592 (39.3)/1,796 (41.6)	0.91 (0.83, 099)	0.95 (0.88, 1.01)
81	1,422 (34.5)/1,586 (36.3)	0.92 (0.84, 1.01)	0.95 (0.88, 1.02)
91	1,444 (35.3)/1,228 (31.7)	0.85 (0.77-0.93)	0.90 (0.83, 0.97)
103	1,214 (30.7)/1,386 (33.8)	0.87 (0.79, 0.95)	0.91 (0.84, 0.98)
128	1,223 (31.7)/1,301 (33.2)	0.94 (0.85, 1.03)	0.96 (0.89, 1.04)
157	730 (20.9)/887 (25.4)	0.77 (0.69, 0.87)	0.82 (0.74, 0.91)
166	908 (26.0)/1,111 (31.5)	0.77 (0.69, 0.85)	0.83 (0.76, 0.90)
**F:M OR (95% CI) per year of age**		**0.98 (0.97, 1.00)**	**0.99 (0.98, 0.99)**
P(interaction) with sex		***0.01***	***0.001***
**Vomiting**			
57	3,255 (71.9)/3,413 (70.0)	1.10 (1.00, 1.20)	1.03 (0.98, 1.08)
69	2,098 (51.7)/2,124 (49.1)	1.11 (1.02, 1.21)	1.05 (0.99, 1.12)
81	1,897 (45.6)/1,907 (43.6)	1.10 (1.01, 1.20)	1.05 (0.99, 1.12)
91	1,691 (43.5)/1,722 (42.2)	1.06 (0.97, 1.15)	1.03 (0.96, 1.10)
103	1,605 (40.5)/1,601 (39.1)	1.06 (0.97, 1.16)	1.04 (0.97, 1.11)
128	1,439 (37.5)/1,441 (36.9)	1.03 (0.94, 1.13)	1.02 (0.95, 1.10)
157	876 (25.0)/884 (25.3)	0.98 (0.88, 1.10)	0.99 (0.90, 1.09)
166	996 (28.5)/960 (27.2)	1.07 (0.96, 1.19)	1.05 (0.95, 1.15)
**F:M OR (95% CI) per year of age**		**0.99 (0.98, 1.00)**	**1.00 (0.99, 1.01)**
P(interaction) with sex		***0.25***	***0.65***
**Cough**			
57	4,248 (93.4)/4,527 (91.8)	1.11 (0.95, 1.30)	1.00 (0.97, 1.05)
69	3,490 (85.5)/3,709 (84.9)	1.05 (0.93, 1.18)	1.01 (0.96, 1.05)
81	3,221 (78.0)/3,346 (76.5)	1.09 (0.98, 1.21)	1.02 (0.97, 1.07)
91	2,961 (75.8)/3,080 (74.8)	1.06 (0.95, 1.17)	1.01 (0.96, 1.07)
103	2,947 (73.6)/3,011 (73.1)	1.02 (0.93, 1.13)	1.01 (0.96, 1.06)
128	2,834 (73.5)/2,790 (71.2)	1.12 (1.02, 1.24)	1.03 (0.98, 1.09)
157	2,380 (67.3)/2,259 (64.1)	1.15 (1.04, 1.27)	1.05 (0.99, 1.11)
166	2,452 (70.0)/2,431 (68.7)	1.07 (0.96, 1.18)	1.02 (0.96, 1.08)
**F:M OR (95% CI) per year of age**		**1.01 (0.99, 1.02)**	**1.00 (1.00, 1.01)**
P(interaction) with sex		***0.27***	***0.05***

Supplementary Table 7 continued

	N (%) outcome females / males	OR (95% CI)	RR (95% CI)
**High temperature**			
57	3,401 (75.2)/3,636 (74.8)	1.02 (0.93, 1.12)	1.01 (0.96, 1.05)
69	2,495 (61.4)/2,670 (61.7)	0.99 (0.91, 1.08)	1.00 (0.94, 1.05)
81	2,26 (53.9)/2,285 (52.3)	1.07 (0.98, 1.16)	1.03 (0.97, 1.09)
91	2,006 (51.4)/2,073 (50.5)	1.04 (0.95, 1.13)	1.02 (0.96, 1.08)
103	1,948 (48.9)/1,970 (47.8)	1.05 (0.96, 1.14)	1.02 (0.96, 1.09)
128	1,889 (49.1)/1,832 (46.8)	1.10 (1.00, 1.20)	1.05 (0.98, 1.12)
157	1,342 (38.1)/1,257 (35.9)	1.10 (1.00, 1.21)	1.06 (0.98, 1.15)
166	1,533 (43.8)/1,428 (40.4)	1.15 (1,05, 1.26)	1.08 (1.01, 1.16)
**F:M OR (95% CI) per year of age**		**1.01 (1.00, 1.02)**	**1.01 (1.00, 1.01)**
P(interaction) with sex		***0.01***	***0.002***
**Earache**			
57	1,916 (42.3)/1,933 (39.6)	1.12 (1.03, 1.21)	1.07 (1.00, 1.14)
69	1,529 (36.6)/1,413 (31.9)	1.24 (1.13, 1.35)	1.15 (1.07, 1.24)
81	1,273 (30.8)/1,103 (25.2)	1.32 (1.20, 1.45)	1.22 (1.13, 1.33)
91	1,165 (29.2)/940 (22.3)	1.44 (1.30, 1.59)	1.31 (1.20, 1.43)
103	1.073 (26.6)/817 (19.5)	1.50 (1.35, 1.66)	1.37 (1.25, 1.50)
128	904 (23.5)/638 (16.2)	1.58 (1.41, 1.77)	1.44 (1.31, 1.60)
157	673 (19.1)/444 (12.7)	1.63 (1.43, 1.85)	1.51 (1.34, 1.70)
166	675 (19.2)/483 (13.6)	1.51 (1.33, 1.72)	1.41 (1.26, 1.59)
**F:M OR (95% CI) per year of age**		**1.04 (1.03, 1.06)**	**1.04 (1.03, 1.05)**
P(interaction) with sex		***<0.001***	***<0.001***
**Ear discharge**			
57	323 (7.2)/375 (7.7)	0.92 (0.79, 1.07)	0.92 (0.80, 1.07)
69	257 (6.2)/285 (6.4)	0.96 (0.80, 1.14)	0.96 (0.81, 1.14)
81	215 (5.2)/243 (5.6)	0.93 (0.77, 1.13)	0.94 (0.78, 1.13)
91	170 (4.3)/170 (4.0)	1.06 (0.86, 1.32)	1.06 (0.86, 1.31)
103	128 (3.2)/129 (3.1)	1.03 (0.80, 1.32)	1.03 (0.81, 1.32)
128	99 (2.6)/81 (2.1)	1.25 (0.93, 1.68)	1.24 (0.93, 1.67)
157	67 (1.9)/67 (1.9)	1.00 (0.71, 1.40)	1.00 (0.71, 1.40)
166	90 (2.3)/83 (2.3)	0.97 (0.71, 1.33)	0.97 (0.72, 1.33)
**F:M OR (95% CI) per year of age**		**1.02 (0.98, 1.05)**	**1.02 (0.98, 1.05)**
P(interaction) with sex		***0.29***	***0.30***

Supplementary Table 7 continued

	N (%) outcome females / males	OR (95% CI)	RR (95% CI)
**Stomach ache**			
57	2,549 (56.2)/2,405 (49.4)	1.31 (1.21, 1.42)	1.14 (1.08, 1.20)
69	2,390 (57.4)/2,243 (50.5)	1.32 (1.21, 1.44)	1.14 (1.07, 1.20)
81	2,607 (63.2)/2,453 (56.1)	1.34 (1.23, 1.46)	1.13 (1.07, 1.19)
91	2,445 (61.3)/2,281 (54.0)	1.34 (1.23, 1.47)	1.13 (1.07, 1.20)
103	2,541 (62.8)/2,294 (54.5)	1.41 (1.29, 1.54)	1.15 (1.09, 1.22)
128	2,450 (63.4)/2.072 (52.8)	1.55 (1.42, 1.70)	1.20 (1.13, 1.27)
157	2,303 (65.2)/1,517 (43.2)	2.46 (2.24, 2.71)	1.51 (1.41, 1.61)
166	2,462 (70.1)/1,640 (46.3)	2.71 (2.46, 2.99)	1.51 (1.42, 1.61)
**F:M OR (95% CI) per year of age**		**1.08 (1.07, 1.09)**	**1.03 (1.03, 1.04)**
P(interaction) with sex		***<0.001***	***<0.001***
**Rash**			
57	1,258 (27.8)/1,320 (27.1)	1.03 (0.94, 1.13)	1.02 (0.95, 1.11)
69	917 (22.0)/911 (20.6)	1.09 (0.98, 1.21)	1.07 (0.98, 1.17)
81	854 (20.7)/742 (17.0)	1.28 (1.14, 1.42)	1.22 (1.11, 1.35)
91	749 (18.8)/638 (15.1)	1.30 (1.16, 1.46)	1.24 (1.12, 1.38)
103	822 (20.3)/631 (15.0)	1.45 (1.29, 1.62)	1.35 (1.22, 1.50)
128	753 (19.5)/574 (14.6)	1.42 (1.26, 1.60)	1.33 (1.20, 1.49)
157	582 (16.6)/439 (12.6)	1.38 (1.21, 1.58)	1.32 (1.16, 1.49)
166	631 (18.0)/445 (12.6)	1.53 (1.34, 1.74)	1.43 (1.27, 1.62)
**F:M OR (95% CI) per year of age**		**1.04 (1.03, 1.06)**	**1.04 (1.03, 1.05)**
P(interaction) with sex		***<0.001***	***<0.001***
**Wheezing**			
57	613 (13.5)/917 (18.8)	0.68 (0.60, 0.76)	0.72 (0.65, 0.80)
69	509 (12.2)/700 (15.8)	0.74 (0.66, 0.84)	0.77 (0.69, 0.87)
81	440 (10.7)/600 (13.7)	0.75 (0.66, 0.86)	0.78 (0.69, 0.88)
91	353 (8.8)/531 (12.6)	0.68 (0.59, 0.78)	0.70 (0.62, 0.81)
103	356 (8.8)/557 (13.2)	0.63 (0.55, 0.73)	0.67 (0.58, 0.76)
128	333 (8.6)/490 (12.5)	0.66 (0.57, 0.77)	0.69 (0.60, 0.79)
157	311 (8.8)/432 (12.4)	0.69 (0.59, 0.80)	0.71 (0.62, 0.83)
166	288 (8.2)/372 (10.5)	0.76 (0.65, 0.90)	0.78 (0.67, 0.91)
**F:M OR (95% CI) per year of age**		**1.00 (0.98, 1.02)**	**1.00 (0.98, 1.01)**
P(interaction) with sex		***0.93***	***0.86***

Supplementary Table 7 continued

	N (%) outcome females / males	OR (95% CI)	RR (95% CI)
**Breathlessness**			
57	304 (6.7)/498 (10.2)	0.63 (0.54, 0.73)	0.66 (0.57, 0.76)
69	255 (6.1)/373 (8.4)	0.71 (0.60, 0.84)	0.73 (0.62, 0.85)
81	219 (5.3)/331 (7.6)	0.68 (0.57, 0.82)	0.70 (0.59, 0.83)
91	188 (4.7)/300 (7.1)	0.65 (0.54, 0.78)	0.66 (0.55, 0.80)
103	249 (0.6)/341 (8.1)	0.74 (0.63, 0.88)	0.76 (0.65, 0.89)
128	237 (6.1)/353 (9.0)	0.66 (0.56, 0.79)	0.68 (0.58, 0.81)
157	273 (7.8)/287 (8.2)	0.94 (0.79, 1.12)	0.95 (0.80, 1.12)
166	252 (7.2)/244 (6.9)	1.05 (0.87, 1.26)	1.04 (0.88, 1.25)
**F:M OR (95% CI) per year of age**		**1.05 (1.03, 1.07)**	**1.04 (1.02, 1.07)**
P(interaction) with sex		***<0.001***	***<0.001***
**Headache**			
57	1,515 (33.3)/1,505 (30.8)	1.12 (1.03, 1.22)	1.08 (1.01, 1.16)
69	1,285 (30.9)/1,291 (29.1)	1.09 (0.99, 1.19)	1.06 (0.98, 1.14)
81	1,694 (41.0)/1,723 (39.4)	1.07 (0.98, 1.17)	1.04 (0.97, 1.11)
91	1,919 (48.1)/1,873 (44.3)	1.17 (1.07, 1.27)	1.09 (1.02, 1.16)
103	2,168 (53.6)/2,142 (50.9)	1.11 (1.02, 1.21)	1.05 (0.99, 1.12)
128	2,669 (69.0)/2,480 (63.1)	1.30 (1.18, 1.43)	1.09 (1.03, 1.15)
157	2,704 (76.2)/2,412 (68.4)	1.48 (1.33, 1.64)	1.11 (1.05, 1.18)
166	2,807 (79.8)/2,496 (70.4)	1.66 (1.49, 1.86)	1.13 (1.07, 1.20)
**F:M OR (95% CI) per year of age**		**1.05 (1.03, 1.06)**	**1.01 (1.00, 1.01)**
P(interaction) with sex		***<0.001***	***0.01***
**Constipation**			
57	698 (15.4)/659 (13.5)	1.16 (1.04, 1.31)	1.14 (1.02, 1.27)
69	466 (11.2)/421 (9.5)	1.20 (1.05, 1.38)	1.18 (1.03, 1.35)
81	490 (11.9)/389 (8.9)	1.38 (1.20, 1.59)	1.33 (1.17, 1.52)
91	439 (11.0)/388 (9.2)	1.22 (1.06, 1.41)	1.20 (1.05, 1.38)
103	438 (10.9)/385 (9.2)	1.21 (1.05, 1.40)	1.19 (1.03, 1.36)
128	410 (10.7)/347 (8.8)	1.23 (1.06, 1.43)	1.21 (1.04, 1.39)
157	301 (8.6)/270 (7.8)	1.12 (0.94, 1.33)	1.11 (0.94, 1.31)
166	277 (7.9)/259 (7.3)	1.09 (0.91, 1.30)	1.08 (0.91, 1.28)
**F:M OR (95% CI) per year of age**		**0.99 (0.97, 1.01)**	**0.99 (0.98, 1.01)**
P(interaction) with sex		***0.39***	***0.45***

Supplementary Table 7 continued

	N (%) outcome females / males	OR (95% CI)	RR (95% CI)
**Head lice / scabies**			
57	843 (18.5)/565 (11.6)	1.74 (1.55, 1.95)	1.60 (1.44, 1.78)
69	1,356 (32.7)/824 (18.7)	2.12 (1.92, 2.34)	1.75 (1.61, 1.91)
81	2,408 (58.3)/1,571 (35.9)	2.50 (2.29, 2.73)	1.62 (1.52, 1.73)
91	2,408 (60.3)/1,546 (36.6)	2.64 (2.41, 2.88)	1.65 (1.55, 1.76)
103	2,427 (60.0)/1.410 (33.5)	2.98 (2.73, 3.26)	1.79 (1.68, 1.91)
128	1,822 (47.1)/770 (19.6)	3.65 (3.30, 4.03)	2.40 (2.21, 2.61)
157	791 (22.5)/313 (8.9)	2.95 (2.57, 3.40)	2.51 (2.21, 2.87)
166	593 (16.9)/270 (7.6)	2.47 (2.12, 2.87)	2.22 (1.92, 2.56)
**F:M OR (95% CI) per year of age**		**1.03 (1.02, 1.04)**	**1.03 (1.02, 1.04)**
P(interaction) with sex		***<0.001***	***<0.001***
**Eczema**			
81	724 (17.5)/639 (14.6)	1.24 (1.11, 1.40)	1.20 (1.08, 1.33)
91	708 (17.8)/632 (15.0)	1.23 (1.09, 1.38)	1.19 (1.07, 1.32)
103	699 (17.3)/580 (13.8)	1.31 (1.16, 1.47)	1.25 (1.12, 1.40)
128	649 (16.8)/543 (13.8)	1.26 (1.11, 1.42)	1.21 (1.08, 1.36)
157	485 (3.8)/422 (12.1)	1.16 (1.01, 1.34)	1.14 (1.00, 1.30)
166	482 (13.8)/432 (11.9)	1.18 (1.02, 1.35)	1.15 (1.01, 1.31)
**F:M OR (95% CI) per year of age**		**0.99 (0.97, 1.01)**	**0.99 (0.98, 1.01)**
P(interaction) with sex		***0.31***	***0.38***
**Asthma**			
81	454 (11.0)/609 (13.9)	0.76 (0.67, 0.87)	0.79 (0.70, 0.89)
91	387 (9.7)/568 (13.4)	0.69 (0.60, 0.79)	0.72 (0.63, 0.82)
103	402 (10.0)/572 (13.7)	0.70 (0.61, 0.80)	0.73 (0.64, 0.83)
128	389 (10.1)/546 (13.9)	0.69 (0.60, 0.79)	0.72 (0.63, 0.82)
157	360 (10.2)/473 (13.5)	0.73 (0.63, 0.84)	0.75 (0.66, 0.87)
166	366 (10.4)/452 (12.8)	0.80 (0.69, 0.92)	0.82 (0.71, 0.94)
**F:M OR (95% CI) per year of age**		**1.01 (0.99, 1.03)**	**1.01 (0.99, 1.02)**
P(interaction) with sex		***0.54***	***0.55***

Supplementary Table 7 continued

	N (%) outcome females / males	OR (95% CI)	RR (95% CI)
**Hay fever**			
81	208 (5.0)/328 (7.5)	0.65 (0.55, 0.78)	0.67 (0.56, 0.80)
91	281 (7.1)/442 (10.5)	0.65 (0.55, 0.76)	0.67 (0.58, 0.78)
103	442 (11.0)/604 (14.5)	0.73 (0.64, 0.84)	0.76 (0.67, 0.86)
128	523 (13.6)/650 (16.6)	0.79 (0.70, 0.89)	0.82 (0.73, 0.92)
157	601 (17.1)/781 (22.4)	0.72 (0.64, 0.81)	0.77 (0.69, 0.85)
166	655 (18.7)/830 (23.4)	0.75 (0.67, 0.84)	0.80 (0.72, 0.88)
**F:M OR (95% CI) per year of age**		**1.01 (0.99, 1.03)**	**1.02 (1.00, 1.03)**
P(interaction) with sex		***0.21***	***0.05***
**Pains in arms / legs**			
57	650 (14.4)/732 (15.1)	0.95 (0.85, 1.07)	0.96 (0.86, 1.07)
69	732 (17.6)/786 (17.8)	0.99 (0.88, 1.10)	0.99 (0.90, 1.09)
81	736 (18.2)/832 (19.5)	0.92 (0.82, 1.03)	0.93 (0.85, 1.03)
103	857 (21.3)/908 (21.8)	0.97 (0.88, 1.08)	0.98 (0.89, 1.07)
140	1,003 (27.4)/1,151 (31.7)	0.82 (0.74, 0.90)	0.87 (0.80, 0.94)
157	1,026 (30.2)/1,173 (34.7)	0.81 (0.74, 0.90)	0.87 (0.80, 0.95)
**F:M OR (95% CI) per year of age**		**0.98 (0.96, 0.99)**	**0.98 (0.97, 1.00)**
P(interaction) with sex		***0.003***	***0.01***
**Food allergy**			
65	349 (8.0)/425 (9.2)	0.86 (0.74, 1.00)	0.87 (0.76, 1.01)
81	373 (9.8)/422 (10.4)	0.93 (0.80, 1.08)	0.94 (0.82, 1.08)
103	224 (5.5)/273 (6.5)	0.85 (0.71, 1.02)	0.85 (0.72, 1.02)
157	341 (9.6)/347 (9.8)	0.97 (0.83, 1.14)	0.98 (0.84, 1.13)
**F:M OR (95% CI) per year of age**		**1.01 (0.99, 1.04)**	**1.01 (0.99, 1.04)**
P(interaction) with sex		***0.23***	***0.23***
**Other allergy**			
65	514 (12.3)/576 (13.0)	0.94 (0.82, 1.06)	0.94 (0.84, 1.06)
81	556 (14.1)/635 (15.3)	0.91 (0.81, 1.03)	0.92 (0.83, 1.04)
103	856 (21.5)/973 (23.5)	0.89 (0.80, 0.99)	0.91 (0.83, 1.00)
157	925 (27.1)/954 (27.9)	0.96 (0.86, 1.07)	0.97 (0.89, 1.06)
**F:M OR (95% CI) per year of age**		**1.00 (0.99, 1.02)**	**1.00 (0.99, 1.02)**
P(interaction) with sex		***0.63***	***0.48***

Supplementary Table 8: OR and RR (95% CI) for infections in females compared with males (cross-sectional samples)

	N (%) outcome females / males	OR (95% CI)	RR (95% CI)
**Chicken pox**			
57	1,723 (38.1)/1,949 (40.2)	0.91 (0.84, 0.99)	0.95 (0.89, 1.01)
69	662 (15.9)/777 (17.6)	0.89 (0.79, 0.99)	0.91 (0.82, 1.00)
81	319 (7.8)/322 (7.4)	1.05 (0.90, 1.24)	1.05 (0.90, 1.22)
91	152 (3.8)/148 (3.5)	1.09 (0.87, 1.37)	1.09 (0.87, 1.36)
103	66 (1.6)/70 (1.7)	0.98 (0.70, 1.38)	0.98 (0.70, 1.37)
128	34 (0.9)/20 (0.5)	1.74 (1.00, 3.02)	1.73 (1.00, 3.00)
166	12 (0.4)/15 (0.4)	0.81 (0.38, 1.74)	0.82 (0.38, 1.74)
**F:M OR (95% CI) per year of age**		**1.07 (1.00, 1.14)**	**1.04 (0.99, 1.10)**
P(interaction) with sex		***0.07***	***0.12***
**Cold sores**			
57	328 (7.7)/319 (6.6)	1.18 (1.01, 1.38)	1.17 (1.00, 1.36)
69	284 (6.9)/259 (5.9)	1.18 (0.99, 1.40)	1.16 (0.98, 1.38)
81	269 (6.5)/265 (6.1)	1.08 (0.91, 1.29)	1.07 (0.91, 1.27)
91	299 (7.5)/254 (6.0)	1.27 (1.06, 1.50)	1.25 (1.05, 1.47)
103	275 (6.8)/233 (5.5)	1.24 (1.04, 1.49)	1.23 (1.03, 1.46)
128	298 (7.9)/221 (5.7)	1.40 (1.17, 1.68)	1.37 (1.15, 1.63)
166	298 (8.5)/188 (5.4)	1.64 (1.36, 1.98)	1.59 (1.32, 1.91)
**F:M OR (95% CI) per year of age**		**1.04 (1.02, 1.07)**	**1.04 (1.02, 1.06)**
P(interaction) with sex		***<0.001***	***0.001***
**Eye infection**			
57	689 (15.3)/736 (15.3)	1.00 (0.90, 1.12)	1.00 (0.90, 1.11)
69	338 (8.2)/349 (7.9)	1.03 (0.88, 1.21)	1.03 (0.89, 1.19)
81	195 (4.7)/188 (4.3)	1.10 (0.90, 1.35)	1.10 (0.90, 1.34)
91	141 (3.5)/134 (3.2)	1.12 (0.88, 1.42)	1.11 (0.88, 1.41)
103	151 (3.7)/110 (2.6)	1.44 (1.13, 1.85)	1.43 (1.12, 1.83)
128	149 (3.9)/131 (3.4)	1.17 (0.92, 1.48)	1.16 (0.92, 1.47)
166	185 (5.3)/1.03 (2.9)	1.87 (1.46, 2.39)	1.82 (1.43, 2.32)
**F:M OR (95% CI) per year of age**		**1.11 (1.06, 1.15)**	**1.10 (1.06, 1.14)**
P(interaction) with sex		***<0.001***	***<0.001***

Supplementary Table 8 continued

	N (%) outcome females / males	OR (95% CI)	RR (95% CI)
**Ear infection**			
57	1,507 (33.4)/1,605 (33.2)	1.01 (0.93, 1.10)	1.01 (0.94, 1.08)
69	1,099 (26.5)/1,103 (25.0)	1.08 (0.98, 1.19)	1.06 (0.97, 1.15)
81	808 (19.6)/773 (17.8)	1.13 (1.02, 1.26)	1.11 (1.00, 1.22)
91	613 (15.4)/535 (12.7)	1.25 (1.10, 1.42)	1.21 (1.08, 1.36)
103	459 (11.3)/385 (9.2)	1.27 (1.10, 1.46)	1.24 (1.08, 1.42)
128	363 (9.5)/255 (6.5)	1.50 (1.27, 1.77)	1.45 (1.24, 1.70)
166	236 (6.8)/212 (6.0)	1.14 (0.94, 1.38)	1.13 (0.94, 1.36)
**F:M OR (95% CI) per year of age**		**1.05 (1.02, 1.07)**	**1.04 (1.02, 1.06)**
P(interaction) with sex		***<0.001***	***<0.001***
**Chest infection**			
57	977 (21.7)/1,190 (24.7)	0.85 (0.77, 0.93)	0.88 (0.81, 0.96)
69	653 (15.9)/771 (17.6)	0.88 (0.79, 0.99)	0.90 (0.81, 1.00)
81	431 (10.5)/ 475 (10.9)	0.96 (0.83, 1.10)	0.96 (0.84, 1.09)
91	220 (5.5)/293 (7.0)	0.78 (0.65, 0.94)	0.79 (0.67, 0.95)
103	193 (4.8)/244 (5.8)	0.81 (0.67, 0.98)	0.82 (0.68, 0.99)
128	192 (5.0)/228 (5.8)	0.85 (0.70, 1.04)	0.86 (0.71, 1.04
166	174 (5.0)/202 (5.7)	0.87 (0.70, 1.07)	0.87 (0.71, 1.07)
**F:M OR (95% CI) per year of age**		**1.00 (0.97, 1.03)**	**1.00 (0.97, 1.02)**
P(interaction) with sex		***0.91***	***0.73***
**Tonsilitis / laryngitis**			
91	320 (8.0)/288 (6.8)	1.19 (1.01, 1.41)	1.18 (1.00, 1.38)
103	299 (7.4)/214 (5.1)	1.49 (1.24, 1.79)	1.45 (1.22, 1.73)
128	348 (9.1)/221 (5.7)	1.67 (1.40, 1.98)	1.61 (1.36, 1.90)
166	362 (10.4)/267 (7.6)	1.41 (1.20, 1.67)	1.37 (1.17, 1.61)
**F:M OR (95% CI) per year of age**		**1.02 (0.99, 1.06)**	**1.02 (0.99, 1.05)**
P(interaction) with sex		***0.24***	***0.27***

Supplementary Table 8 continued

	N (%) outcome females / males	OR (95% CI)	RR (95% CI)
**Influenza**			
91	223 (5.6)/214 (5.1)	1.11 (0.91, 1.34)	1.10 (0.91, 1.33)
103	167 (4.1)/173 (4.1)	1.00 (0.81, 1.25)	1.00 (0.81, 1.24)
128	196 (5.1)/176 (4.5)	1.14 (0.92, 1.40)	1.13 (0.92, 1.39)
166	235 (6.8)/230 (6.5)	1.04 (0.86, 1.26)	1.04 (0.87, 1.25)
**F:M OR (95% CI) per year of age**		**1.00 (0.96, 1.04)**	**1.00 (0.96, 1.04)**
P(interaction) with sex		***0.88***	***0.88***
**Cold**			
57	4,406 (97.0)/4,687 (96.1)	1.31 (1.04, 1.63)	1.01 (0.97, 1.05)
69	3,805 (93.0)/4,014 (91.7)	1.20 (1.02, 1.41)	1.01 (0.97, 1.06)
81	3,639 (88.2)/3,774 (86.3)	1.18 (1.04, 1.34)	1.02 (0.98, 1.07)
91	2,687 (93.7)/3,814 (91.9)	1.31 (1.11, 1.56)	1.02 (0.97, 1.07)
103	3,695 (91.1)/3,773 (89.6)	1.19 (1.03, 1.38)	1.02 (0.97, 1.06)
128	3,174 (82.2)/3,036 (77.6)	1.34 (1.20, 1.50)	1.06 (1.01, 1.11)
166	3.022 (86.2)/2,905 (82.0)	1.36 (1.20, 1.55)	1.05 (1.00, 1.11)
**F:M OR (95% CI) per year of age**		**1.02 (1.00, 1.03)**	**1.01 (1.00, 1.01)**
P(interaction) with sex		***0.07***	***<0.001***
**Urinary infection**			
57	400 (8.9)/192 (4.0)	2.35 (1.97, 2.81)	2.23 (1.88, 2.65)
69	271 (6.5)/117 (2.7)	2.56 (2.05, 3.19)	2.46 (1.98, 3.05)
81	224 (5.4)/93 (2.1)	2.64 (2.06, 3.37)	2.55 (2.00, 3.25)
91	172 (4.3)/75 (1.8)	2.50 (1.90, 3.29)	2.43 (1.85, 3.19)
103	176 (4.4)/55 (1.3)	3.44 (2.53, 4.67)	3.33 (2.46, 4.51)
128	138 (3.6)/41 (1.1)	3.52 (2.48, 5.00)	3.43 (2.42, 4.86)
166	118 (3.4)/24 (0.7)	5.13 (3.30, 7.98)	4.99 (3.22, 7.74)
**F:M OR (95% CI) per year of age**		**1.10 (1.05, 1.16)**	**1.10 (1.05, 1.16)**
P(interaction) with sex		***<0.001***	***<0.001***

Supplementary Table 8 continued

	N (%) outcome females / males	OR (95% CI)	RR (95% CI)
**Worm infection**			
57	477 (10.5)/397 (8.1)	1.33 (1.15, 1.53)	1.29 (1.13, 1.48)
69	418 (10.1)/318 (7.2)	1.45 (1.24, 1.68)	1.40 (1.21, 1.62)
81	473 (11.5)/340 (7.8)	1.54 (1.33, 1.78)	1.47 (1.28, 1.69)
91	407 (10.2)/313 (7.4)	1.42 (1.22, 1.66)	1.38 (1.19, 1.60)
103	390 (9.7)/267 (6.4)	1.58 (1.34, 1.85)	1.52 (1.30, 1.78)
128	237 (6.1)/144 (3.7)	1.72 (1.39, 2.12)	1.67 (1.36, 2.06)
157	81 (2.3)/66 (1.9)	1.22 (0.88, 1.70)	1.21 (0.88, 1.69)
166	55 (1.6)/62 (1.8)	0.90 (0.62, 1.29)	0.90 (0.62, 1.29)
**F:M OR (95% CI) per year of age**		**0.99 (0.97, 1.02)**	**1.00 (0.98, 1.02)**
P(interaction) with sex		***0.61***	***0.84***
